# Postoperative Complications and Long-Term Quality of Life After Multimodality Treatment for Esophageal Cancer: An Analysis of the Prospective Observational Cohort Study of Esophageal-Gastric Cancer Patients (POCOP)

**DOI:** 10.1245/s10434-021-10144-5

**Published:** 2021-05-25

**Authors:** E. Jezerskyte, M. I. van Berge Henegouwen, H. W. M. van Laarhoven, J. J. van Kleef, W. J. Eshuis, J. Heisterkamp, H. H. Hartgrink, C. Rosman, R. van Hillegersberg, M. C. C. M. Hulshof, M. A. G. Sprangers, S. S. Gisbertz, on behalf of the Dutch UpperGI Cancer Group

**Affiliations:** 1 Amsterdam UMC, Department of Surgery, Cancer Center Amsterdam, University of Amsterdam, Amsterdam, The Netherlands; 2 Amsterdam UMC, Department of Medical Oncology, Cancer Center Amsterdam, University of Amsterdam, Amsterdam, The Netherlands; 3 Department of Surgery, Embraze Comprehensive Cancer Network, Elisabeth- Tweesteden Hospital, Tilburg, The Netherlands; 4 Department of Surgery, Leiden University Medical Center, Leiden, The Netherlands; 5 Department of Surgery, Radboud University Medical Center, Nijmegen, The Netherlands; 6 Department of Surgery, University Medical Center Utrecht, Utrecht, The Netherlands; 7 Amsterdam UMC, Department of Radiotherapy, Cancer Center Amsterdam, University of Amsterdam, Amsterdam, The Netherlands; 8 Amsterdam UMC, Department of Medical Psychology, Cancer Center Amsterdam, University of Amsterdam, Amsterdam, The Netherlands

## Abstract

**Background:**

Esophagectomy has major effects on health-related quality of life (HR-QoL). Postoperative complications might contribute to a decreased HR-QOL. This population-based study aimed to investigate the difference in HR-QoL between patients with and without complications after esophagectomy for cancer.

**Methods:**

A prospective comparative cohort study was performed with data from the Netherlands Cancer Registry (NCR) and Prospective Observational Cohort Study of Esophageal-Gastric Cancer Patients (POCOP). All patients with esophageal and gastroesophageal junction (GEJ) cancer after esophagectomy in the period 2015–2018 were enrolled. The study investigated HR-QoL at baseline, then 3, 6, 9, 12, 18, and 24 months postoperatively, comparing patients with and without complications as well as with and without anastomotic leakage.

**Results:**

The 486 enrolled patients comprised 270 patients with complications and 216 patients without complications. Significantly more patients with complications had comorbidities (69.6% vs 57.3%; *p *= 0.001). No significant difference in HR-QoL was found over time between the patients with and without complications. In both groups, a significant decline in short-term HR-QoL was found in various HR-QoL domains, which were restored to the baseline level during the 12-month follow-up period. No significant difference was found in HR-QoL between the patients with and without anastomotic leakage. The patients with grades 2 and 3 anastomotic leakage reported significantly more “choking when swallowing” at 6 months (*ß* = 14.5; 95% confidence interval [CI], − 24.833 to − 4.202; *p *= 0.049), 9 months (*ß* = 22.4, 95% CI, − 34.259 to − 10.591; *p* = 0.007), and 24 months (*ß *= 24.6; 95% CI, − 39.494 to − 9.727; *p *= 0.007) than the patients with grade 1 or no anastomotic leakage.

**Conclusion:**

In general, postoperative complications were not associated with decreased short- or long-term HR-QoL for patients after esophagectomy for esophageal or GEJ cancer. The temporary decrease in HR-QoL likely is related to the nature of esophagectomy and reconstruction itself.

**Supplementary Information:**

The online version contains supplementary material available at 10.1245/s10434-021-10144-5.

Curative treatment for patients with esophageal cancer usually consists of (neo)adjuvant chemo(radio) therapy and surgery. These treatments often are accompanied by side effects and complications.^1^,^2^ Surgeons strive to improve postoperative results by prehabilitation, Enhanced Recovery After Surgery (ERAS) programs, and minimally invasive surgery.^3^–^5^ However, more than 60% of esophagectomy patients still experience postoperative complications.^1^,^6^ A complicated postoperative course often is accompanied by an increase in anxiety and depression, impeding patients’ recovery from surgery.^7^,^8^ Complications also are related to a decreased survival.^9^

Several studies have investigated the impact of postoperative complications on health-related quality of life (HR-QoL) for cancer patients.^10^,^11^ Overall, cancer patients were found to report worse long-term HR-QoL after postoperative complications. A systematic review and meta-analysis have been performed encompassing 50 studies investigating the impact of complications on long-term HR-QoL after cardiac, thoracic, gastrointestinal (GI), and vascular surgery. A negative effect of postoperative complications on patients’ HR-QoL 12 months after operation was found.^11^

Few studies have investigated long-term HR-QoL of patients with and without complications after an esophagectomy.^12^–^15^ Overall, an impaired short- and long-term HR-QoL has been reported by patients with postoperative complications versus patients without postoperative complications. Also, the occurrence of anastomotic leakage has been associated with worse short-term HR-QoL.^15^ However, these studies either did not include a baseline measurement, were performed before the implementation of minimally invasive surgery, did not include information on (neo)adjuvant treatment, or were conducted in a single center with a limited number of patients.^12^–^15^

This study aimed to investigate the difference in short- and long-term HR-QoL for patients with and without a complicated postoperative course after multimodality treatment for esophageal and gastroesophageal junction (GEJ) cancer in a nationwide cohort. We hypothesized that postoperative complications negatively influence short- and long-term HR-QoL.

## Methods

### Study Design

A population-based prospective comparative cohort study was performed with data from the Prospective Observational Cohort Study of Esophageal-Gastric Cancer Patients (POCOP) study and the Netherlands Cancer Registry (NCR).

### Prospective Observational Cohort Study of Esophageal-Gastric Cancer Patients Database

The POCOP is a nationwide Dutch, population-based, observational cohort study of patient-reported outcome measures data from cancer patients, including those with esophageal or gastric cancer. The POCOP aims to gain insight into the quality-of-life course experienced by cancer patients.^16^ The inclusion of patients started in December 2015 in AMC, and during the period from 2016 to 2019, an additional 53 medical centers joined the POCOP study. All the patients with esophageal or gastric cancer in the 54 participating medical centers are asked to participate in the POCOP study irrespective of whether they receive curative treatment or palliative treatment.

Among other forms, the patients in the study complete the validated European Organization for Research and Treatment of Cancer (EORTC) quality-of-life questionnaires at baseline before initiation of treatment, after 3, 6, 9, 12, 18, and 24 months, then annually thereafter.^17^ The POCOP study included 261 patients in 2016, 741 patients in 2017, 1423 patients in 2018, and 2065 patients in 2019. The rationale and design of the POCOP study have been described elsewhere.^16^

The inclusion criteria for the POCOP specified patients with a diagnosis of esophageal or gastric cancer. For the current study, the inclusion criteria specified patients with esophageal and GEJ cancer who underwent an esophagectomy during the period of 2015–2018. The exclusion criteria ruled out patients who underwent surgery for recurrent disease, patients who underwent salvage or palliative surgery, patients with a recurrence, patients undergoing a colon or jejunal interposition, and patients who had no reconstruction performed or required emergency surgery.

Informed consent was collected by the POCOP, and the Privacy Review Board of NCR approved this study. The POCOP study adheres to the required rules and regulations.^16^ Ethical approval for this study was not required under Dutch law. This manuscript was composed using the STROBE checklist.^18^

### The Netherlands Cancer Registry

The NCR manages data from all cancer patients in the Netherlands. This database stores patient, tumor, and treatment information such as gender, age at diagnosis, tumor type and stage, diagnostic data, information on (neo)adjuvant treatment and surgery, postoperative morbidity and mortality, and the hospital in which the patient was treated. All hospitals are required by Dutch law to provide this information to the NCR. The NCR does not register the severity of postoperative complications (Clavien-Dindo grade), nor does it subdivide pulmonary morbidity into separate pulmonary complications. The clinical outcome data of the NCR cancer patients were combined with the POCOP patient-reported outcome measures for research purposes.

### Multimodality Treatment Including Esophagectomy with Curative Intent

The patients with an advanced (≥cT2N0 or cT1N+) esophageal or GEJ carcinoma usually have been treated with chemoradiotherapy according to the CROSS (ChemoRadiotherapy for Oesophageal cancer Followed by Surgery Study) scheme.^19^ In selected cases (e.g.,  > 2 cm involvement of the stomach), perioperative chemotherapy (previously the MAGIC (Medical Research Council Adjuvant Gastric Infusional Chemotherapy trial), and increasingly during this study period, the FLOT scheme (Fluorouracil, Leucovorin, Oxaliplatin and doceTaxel)) has been administered.^20^,^21^ After neoadjuvant therapy, a transthoracic or transhiatal esophagectomy with a one- or two-field lymphadenectomy and gastric conduit reconstruction using a cervical or intrathoracic anastomosis has been performed by an open, minimally invasive, or hybrid approach.

### Postoperative Complications

The postoperative complications included in the NCR database comprise pulmonary complications, anastomotic leakage, cardiovascular complications, chyle leakage, wound abscess or infection, recurrent laryngeal nerve palsy, thromboembolic complication, and other neurologic complications. Pneumonia was defined as a new or progressive lung infiltration confirmed by radiologic imaging in combination with at least two of the following clinical manifestations: leukocytosis or leukopenia, fever ( > 38 °C), and purulent secretion.^22^ Anastomotic leakage was divided into grades 1–3 according to the Esophageal Complications Consensus Group (ECCG)^23^ as follows: grade 1 (a leakage without the need for a therapy change except for dietary changes), grade 2 (a local leakage requiring an intervention other than surgery), and grade 3 (a leakage requiring surgery). The severity of other complications, such as those categorized by the Clavien-Dindo classification,^24^,^25^ is not registered in the NCR database. The definitions of the postoperative complications used in the NCR can be found in Table S1.

### Outcomes: Quality of Life According to EORTC Questionnaires

The validated cancer-specific European Organisation of Research and Treatment of Cancer (EORTC) QLQ-C30 and the tumor-specific EORTC QLQ-OG25 questionnaires were used for this study.^26^,^27^ The EORTC QLQ-C30 HR-QoL domains were global health (calculated from two questions with response categories ranging from 1 (very poor) 7 (excellent), five functioning scales (physical, role, social, and cognitive and emotional functioning) calculated from 15 questions with response categories ranging from 1 (not at all) to 4 (very much), and scores for nine symptoms (fatigue, nausea and vomiting, pain, dyspnea, insomnia, appetite loss, constipation, diarrhea, and financial difficulties) calculated from 13 questions with response categories ranging from 1 (not at all) to 4 (very much).^26^

The EORTC QLQ-OG25 questionnaire contains 25 questions assessing 16 HR-QoL domains (body image, reflux, dysphagia, pain and discomfort, odynophagia, anxiety, problems with eating, problems with eating with others, trouble with swallowing of saliva, dry mouth, trouble with taste, choking when swallowing, trouble with talking, trouble with coughing, worrying about weight loss, and problems with hair loss). All the questions of the EORTC QLQ-OG25 questionnaire had response categories ranging from 1 (not at all) to 4 (very much).^27^ The 31 HR-QoL domain scores were linearly transformed into scores ranging from 0 to 100. Missing data were managed according to the EORTC scoring manual.^26^,^27^ According to this scoring, a higher score in the global health and functioning domains represents better global health and functioning, and a higher score in the symptom domains represents more symptomatology.

### Statistical Analysis

The chi-square test and Fisher's exact test were used for categorical variables to compare baseline characteristics between the groups. The Shapiro-Wilk test was used to check the distribution pattern of continuous variables. For continuous variables, a Mann-Whitney *U* test was used if the variable was not normally distributed (median with an interquartile range [IQR]), and Student’s *t* test was used if the variable was normally distributed (mean ± standard deviation).

To examine the difference in HR-QoL over time between patients with and without postoperative complications after an esophagectomy, linear mixed-models analysis was performed. To correct for multiple testing, a Bonferroni correction was performed by multiplying the *p* value by the number of tests performed. If a *p* value lower than 0.05 was reached after linear mixed-models analysis and correction for multiple testing, a univariable linear regression analysis was performed for each follow-up time separately to determine the follow-up point at which the difference in HR-QoL between the patients with and without postoperative complications was significant. We did not perform multivariable analyses to adjust for possible a priori differences because our goal was to investigate the difference in HR-QoL in a naturally occurring population.

Univariable linear regression analysis and Bonferroni correction for multiple testing were performed to examine the change in HR-QoL between baseline, short-term (3, 6, and 9 months), and long-term (12, 18, and 24 months) follow-up evaluations for patients with postoperative complications and patients without postoperative complications separately.

A subgroup analysis was performed to compare the HR-QoL between patients with and without anastomotic leakage, and between patients with grade 2 or 3 anastomotic leakage and those with grade 1 or no anastomotic leakage over time using linear mixed-models analyses. In addition, a separate analysis was performed to investigate the HR-QoL for patients with either a cervical or an intrathoracic anastomosis. Given the small number of patients with anastomotic leakage (*n *= 83) and grade 2 or 3 anastomotic leakage (*n *= 54), a stringent *p* value lower than 0.001 was chosen as statistically significant in the linear mixed-models analysis. A *p* value lower than 0.05 was chosen as statistically significant in all other analyses.

Because the minimally important change in mean scores representing clinical relevance varies between HR-QoL domains,^26^,^28^ a cutoff point of 10 points is most likely the upper bound for most HR-QoL domains. Therefore, in the current study, a mean HR-QoL score difference or change of more than 10 points was considered clinically relevant.

## Results

### Patient and Tumor Characteristics

The study enrolled 486 patients after an esophagectomy (Table 1). The response rate of the POCOP study was 69.6% at baseline and decreased to 12.5% at 24 months. However, these percentages were based on all the included patients (i.e., the patients undergoing palliative treatment, definitive chemoradiotherapy, primary surgery, or neoadjuvant treatment and surgery). The decrease in response rate was partially attributable to the death of part of this patient population. The exact response rate of the current study population at baseline could not be calculated because such detailed information was not registered separately. However, compared with baseline, the response rates of the current study population were 81.9% at 3 months, 77.7% at 6 months, 68.1% at 9 months, 60.9% at 12 months, 42.0% at 18 months, and 23.3% at 24 months of follow-up evaluation.

**Table 1:** Baseline patient, treatment, and tumor characteristics

	No postoperative complications		Postoperative complications		*p* value^A^
	*n* = 216 *n* (%)		*n* = 270 *n* (%)		
Median age: years (IQR)	66	(60–70)	66	(60–71)	0.297
Gender					
Male	172	(79.6)	216	(80.0)	0.919
Comorbidities					
No	79	(42.7)	79	(30.4)	**0.001**
Yes	106	(57.3)	181	(69.6)	
1 or 2 comorbidities	60	(32.4)	75	(28.8)	
> 2 comorbidities	46	(24.9)	106	(40.8)	
Missing	31	–	10	–	
Cancer	16	(7.4)	31	(11.5)	0.268
Cardiovascular	39	(18.1)	73	(27.0)	0.094
Pulmonal	12	(5.6)	40	(14.8)	**0.004**
Hypertension	59	(27.3)	100	(37.0)	0.154
Cerebrovascular accident	6	(2.8)	9	(3.3)	0.900
Mental	2	(0.9)	6	(2.2)	0.478
Gastrointestinal	7	(3.2)	13	(4.8)	0.542
Liver	0	–	4	(1.5)	0.145
Kidney	4	(1.9)	5	(1.9)	1.000
Rheumatism	2	(0.9)	8	(3.0)	0.206
Infectious disease	0	–	3	(1.1)	0.270
Diabetes	19	(8.8)	42	(15.6)	0.075
ASA classification					
1	13	(6.0)	19	(7.0)	0.108
2	139	(64.4)	163	(60.4)	
3	40	(18.5)	78	(28.9)	
4	1	(0.5)	1	(0.4)	
Missing	23	(10.6)	9	(3.3)	
Systemic chemotherapy					
No	6	(2.8)	10	(3.7)	0.519
Preoperative	195	(90.3)	247	(91.5)	
Pre- and postoperative	15	(6.9)	13	(4.8)	
Radiotherapy					
No	13	(6.0)	25	(9.3)	0.174
Preoperative	202	(93.5)	245	(90.7)	
Postoperative	1	(0.5)	0	0	
Surgical technique					
Open	26	(12.0)	14	(5.2)	**0.008**
Minimally invasive abdomen	9	(4.2)	11	(4.1)	
Minimally invasive thorax	6	(2.8)	7	(2.6)	
Minimally invasive total	154	(71.3)	234	(86.7)	
Missing	21	(9.7)	4	(1.5)	
Surgical approach					
Transthoracic	176	(81.5)	250	(92.6)	**<0.001**
Transhiatal	40	(18.5)	20	(7.4)	
Location anastomosis					
Cervical	45	(20.8)	102	(37.8)	**0.002**
Intrathoracic	142	(65.7)	166	(61.5)	
Unknown	29	(13.4)	2	(0.7)	
cT					
Tx	14	(6.5)	12	(4.4)	0.155
Tis	–	–	1	(0.4)	
T1	4	(1.8)	4	(1.5)	
T2	79	(26.6)	81	(30.0)	
T3	118	(54.6)	165	(61.1)	
T4	1	(0.5)	5	(1.9)	
cN					
N0	110	(50.9)	116	(43.0)	0.074
N1	68	(31.5)	105	(38.9)	
N2	38	(17.6)	42	(15.6)	
N3	0	–	4	(1.5)	
cM					
cM1	3	(1.4)	6	(2.2)	0.737
Histologic type					
Adenocarcinoma	152	(70.4)	178	(65.9)	0.554
Squamous cell carcinoma	46	(21.3)	64	(23.7)	
Other	18	(8.3)	28	(10.4)	
(y)pT					
T0	52	(24.1)	70	(25.9)	0.446
Tx	3	(1.4)	1	(0.4)	
T1	44	(20.3)	40	(14.8)	
T2	38	(17.6)	51	(18.9)	
T3	79	(36.6)	104	(38.5)	
T4	–	–	4	(1.5)	
(y)pN					
N0	124	(57.4)	169	(62.6)	0.535
N1	57	(26.4)	56	(20.7)	
N2	28	(13.0)	35	(13.0)	
N3	7	(3.2)	9	(3.3)	
c/(y)pM					
M1	7	(3.2)	7	(2.6)	1.000
Radicality					
R0	197	(91.2)	252	(93.3)	0.310
R1	8	(3.7)	16	(5.9)	
Unknown	11	(5.1)	2	(0.7)	
Median no. of lymph nodes (IQR)	23	(17.3–32)	24	(20–33)	0.099
Median no. of lymph node metastases (IQR)	0	(0–2)	0	(0–1)	0.360
Tumor response after neoadjuvant therapy					
Complete regression	49	(22.7)	71	(26.3)	0.608
Subtotal pathologic response	44	(20.4)	50	(18.5)	
Partial pathologic response	85	(39.4)	113	(41.9)	
No pathologic response	12	(5.6)	23	(8.5)	
Missing	26	(12.0)	13	(4.8)	

*IQR* interquartile range; *ASA* American Society of Anaesthesiologists classification

^A^Bold *p* values represent significance (*p *< 0.05).

The majority of the included patients in this study were male (79.8%), and the median age was 66 years (IQR, 60–70 years). Most of the patients were treated with neoadjuvant therapy (90.9%). Postoperative complications occurred for 55.6% (*n *= 270) of all the patients (Table 2). Among the most frequent complications were pulmonary complications (22.6%), anastomotic leakage (17.1%), and cardiac complications (11.3%). Of patients with anastomotic leakage, 27.7% had grade 1, 41% had grade 2, 24.1% had grade 3, and 7.2% had an unknown grade anastomotic leakage.

**Table 2:** Postoperative complications of 486 patients with esophageal and gastroesophageal junction cancer after an esophagectomy in the period 2015–2018

	Patients *n* (%)	
All postoperative complications	270	(55.6)
Pulmonary complication	110	(22.5)
Anastomotic leakage^A^	83	(17.1)
Grade 1	23	(27.7)
Grade 2	34	(41.0)
Grade 3	20	(24.1)
Grade unknown	6	(7.2)
Cardiovascular complication	55	(11.3)
Chyle leakage	43	(8.8)
Wound abscess/infection	22	(4.5)
Recurrent laryngeal nerve palsy	13	(2.7)
Thromboembolic complication	4	(0.8)
Other neurologic complication	4	(0.8)

^A^Anastomotic leakage grade 1 (treatment involving observation, medical therapy, or dietary modification), grade 2 (treatment involving nonsurgical intervention), grade 3 (treatment requiring surgical intervention)

The patients with complications had significantly more comorbidities in general (69.6% vs 57.3%; *p* = 0.001) and pulmonary comorbidities in particular (14.8% vs 5.6%; *p *= 0.004). Significantly more minimally invasive esophagectomies (86.7% vs 71.3%; *p *= 0.008) and more cervical anastomoses (37.8% vs 20.8%; *p *= 0.002) were performed in the group with postoperative complications. The patient, treatment and tumor characteristics are presented in Table 1.

### Comparison of HR-QoL Between the Patients with and Without Postoperative Complications

After linear mixed-models analyses and Bonferroni correction for multiple testing, none of the HR-QoL domains were found to differ significantly at baseline or 3, 6, 9, 12, 18, and 24 months after operation between the patients with and without postoperative complications (Tables 3 and S2).

**Table 3:** Linear mixed-models analysis of health-related quality of life (HR-QoL) scores at baseline and at 3-, 6-, 9-, 12-, 18-, and 24-month follow-up visits for patients with (+) and without (−) postoperative complications after esophagectomy

	Mean HR-QoL score^A^							
	Baseline	3 Months	6 Months	9 Months	12 Months	18 Months	24 Months	Corrected *p* value^B^
*N* (+)	270	212	196	171	152	105	50	
*N* (−)	216	187	182	162	145	99	63	
EORTC QLQ-C30								
Global health								
** +**	74.5	71.4	68.3	72.6	72.0	71.9	73.5	10.633
** −**	74.3	70.2	69.2	73.1	74.6	74.7	76.6	
Functioning scores								
Physical functioning								
** +**	88.2	77.6	74.4	78.3	79.4	79.7	80.6	2.263
** −**	88.7	79.1	77.9	82.3	81.9	83.4	82.7	
Role functioning								
** +**	80.5	67.3	61.4	69.2	74.0	76.1	73.7	10.664
** −**	81.9	66.9	65.1	74.2	75.8	76.6	74.8	
Emotional functioning								
** +**	78.7	81.4	83.0	82.8	82.7	81.3	82.8	5.983
** −**	78.0	82.1	84.7	84.4	85.5	85.1	86.3	
Cognitive functioning								
** +**	89.3	85.7	84.5	85.9	84.7	84.4	83.5	21.297
** −**	89.0	86.1	86.2	83.5	86.5	85.9	85.2	
Social functioning								
** +**	86.1	77.1	73.0	76.7	83.2	80.5	85.3	20.584
** –**	83.3	75.8	75.2	80.7	84.3	83.4	84.4	
Symptom scores								
Fatigue								
** +**	26.2	34.1	39.4	35.1	32.6	31.9	31.0	10.323
** −**	25.5	36.8	36.1	31.7	29.6	29.8	29.3	
Nausea and vomiting								
** +**	11.0	10.3	17.6	16.1	12.5	9.9	9.6	18.569
** −**	11.2	13.1	17.2	12.9	8.8	9.8	9.7	
Pain								
** +**	16.4	20.5	19.7	17.8	17.1	16.1	18.0	5.394
** −**	14.7	20.4	17.4	15.8	15.4	13.2	13.7	
Dyspnea								
** +**	16.4	20.5	19.7	17.8	17.1	16.1	18.0	0.651
** −**	14.7	20.4	17.4	15.8	15.4	13.2	13.7	
Insomnia								
** +**	22.8	25.7	23.6	19.1	20.6	21.3	24.9	25.420
** −**	24.3	29.1	23.1	20.4	23.5	22.5	18.2	
Appetite loss								
** +**	18.0	22.8	34.6	22.8	19.7	15.7	12.4	4.185
** −**	18.7	27.0	36.0	24.9	19.8	20.7	18.6	
Constipation								
** +**	14.1	14.3	12.0	9.4	10.2	9.3	11.9	23.436
** −**	13.5	13.6	10.7	12.3	8.9	8.6	10.7	
Diarrhea								
** +**	8.2	12.2	21.5	17.4	15.4	14.7	12.7	23.343
** −**	4.9	11.4	22.2	17.7	16.2	14.6	18.3	
Financial difficulties								
** +**	5.8	7.6	8.2	8.1	7.9	8.3	6.8	13.516
** −**	6.3	8.2	6.2	7.4	7.3	6.1	4.1	
EORTC QLQ-OG25								
Functioning scores								
Body image								
** +**	90.5	85.1	85.7	86.8	84.8	84.2	88.3	15.469
** −**	91.2	87.2	83.1	87.6	86.7	87.7	89.6	
Symptom scores								
Dysphagia								
** +**	21.5	17.5	23.7	17.2	15.4	14.7	8.9	3.379
** −**	21.5	19.8	18.4	13.9	12.9	8.7	9.3	
Eating								
** +**	30.8	28.4	40.6	32.9	31.4	28.1	23.4	22.072
** −**	32.2	31.2	38.5	31.0	27.2	25.2	25.8	
Reflux								
** +**	7.2	6.4	12.8	14.6	14.8	16.1	13.4	8.029
** −**	6.0	8.6	14.9	16.0	16.5	19.0	15.7	
Odynophagia								
** +**	24.4	14.3	16.2	12.8	11.2	12.8	8.3	9.610
** –**	23.9	16.8	15.1	12.2	10.6	6.8	6.8	
Pain and discomfort								
** +**	17.4	9.5	15.0	16.1	15.3	13.7	14.0	24.118
** −**	16.4	14.1	15.3	16.2	15.9	14.3	11.5	
Anxiety								
** +**	48.8	41.2	32.5	30.0	31.4	29.1	28.3	12.245
** −**	50.9	40.4	31.7	29.2	26.5	26.0	24.7	
Eating with others								
** +**	14.0	10.4	16.9	12.5	10.8	11.1	10.7	18.972
** −**	15.0	11.3	13.3	12.7	11.7	9.9	6.8	
Dry mouth								
** +**	13.9	20.5	26.1	21.7	20.7	21.0	18.8	20.553
** −**	12.3	18.9	25.0	19.8	18.7	18.2	23.8	
Trouble with taste								
** +**	12.4	22.2	26.0	18.6	15.3	13.8	17.4	6.913
** −**	9.4	19.6	22.4	15.7	16.9	13.6	12.8	
Trouble swallowing saliva								
** +**	9.5	14.9	14.3	13.2	15.2	15.8	11.7	15.469
** −**	8.8	12.8	16.9	12.4	13.3	12.3	10.4	
Choked when swallowing								
** +**	7.7	8.0	14.3	12.8	11.6	12.9	12.1	0.744
** −**	6.8	8.6	11.5	8.1	7.1	8.4	8.8	
Trouble with coughing								
** +**	21.9	27.5	44.1	36.0	27.7	30.3	27.6	4.123
** −**	19.2	29.5	41.3	31.5	27.1	26.1	21.7	
Trouble talking								
** +**	4.8	9.7	15.5	10.9	9.4	8.8	4.9	1.395
** −**	4.3	10.4	12.6	6.1	6.7	5.9	2.3	
Weight loss								
** +**	17.6	17.7	22.7	24.7	20.6	16.6	19.9	21.049
** −**	18.7	19.6	24.2	20.6	19.9	16.2	15.4	
Problems with hair loss								
** +**	81.7	46.6	54.9	61.0	62.3	54.8	69.9	16.368
** −**	73.3	47.4	57.1	49.7	57.5	62.4	59.3	

*N* (+), number of patients with postoperative complications; *N* (−), number of patients without postoperative complications

^A^Values are represented as mean HR-QoL scores.

^B^Corrected *p* value = *p* value over time that is corrected for multiple testing according to Bonferroni method. Bold *p* value represents significance (*p *< 0.05).

### Change in HR-QoL for the Patients with No Postoperative Complications

**Fig. 1:**
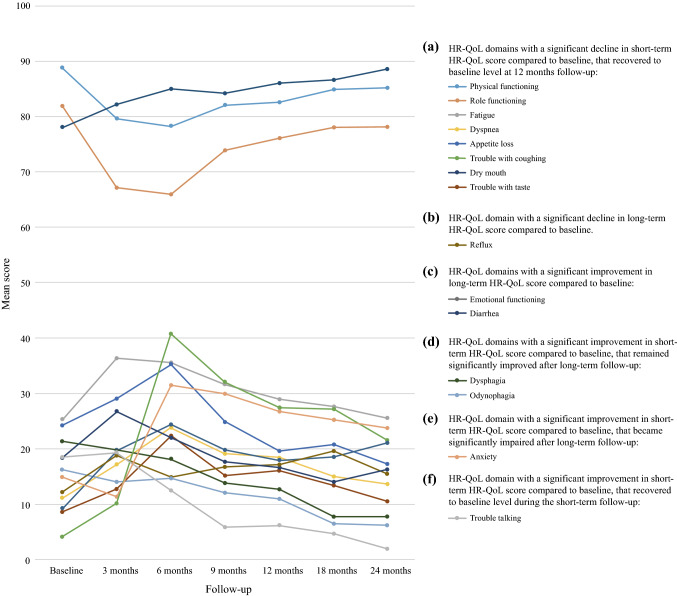
Change in health-related quality of life (HR-QoL) for patients without postoperative complications. **A** A significant decline in short-term HR-QoL score compared with baseline was found in the following eight HR-QoL domains, which had recovered to baseline level at the 12-month follow-up visit: “physical functioning” (at 3, 6, and 9 months, *p *< 0.001), “role functioning” (at 3 and 6 months, *p *< 0.001), “fatigue” (at 3 and 6 months, *p* = 0.002), “trouble with coughing” (at 3 months, *p *= 0.047; at 6 and 9 months, *p *≤ 0.001), “dyspnea” (at 6 months, *p* < 0.001), “appetite loss” (at 6 months, *p *< 0.001), “dry mouth” (at 6 months, *p* = 0.004), and “trouble with taste” (at 3 months, *p *= 0.005; at 6 months, *p *< 0.001). **B**,**C** In three HR-QoL domains, the HR-QoL score either remained significantly impaired (“reflux,” *p *= 0.001) or improved significantly (“emotional functioning,” *p *= 0.003; “diarrhea,” *p *< 0.001) during a long-term follow-up period compared with baseline. **D**,**E**,**F** In four HR-QoL domains, the short-term HR-QoL score improved compared with baseline, and remained significantly improved (“dysphagia” and “odynophagia,” *p* ≤ 0.001) or became significantly impaired (“anxiety,” *p* < 0.001) during the long-term follow-up period, or recovered to baseline level during the short-term follow-up period (“trouble talking,” *p *= 0.001).

A univariable linear regression analysis of the HR-QoL domains was performed between baseline and the 3-, 6-, 9-, 12-, 18-, and 24-month follow-up evaluations for the patients without postoperative complications (Table S3). In eight HR-QoL domains (“trouble with coughing” [at 6 and 9 months], “role functioning,” “fatigue” and “trouble with taste” [at 3 and 6 months], “physical functioning,” “dyspnea,” “appetite loss,” and dry mouth [at 6 months]), a significant and clinically relevant decline in short-term HR-QoL scores compared with baseline was found, which had recovered to the baseline level at the 12-month follow-up evaluation (Fig. 1).

### Change in HR-QoL for the Patients with Postoperative Complications

**Fig. 2:**
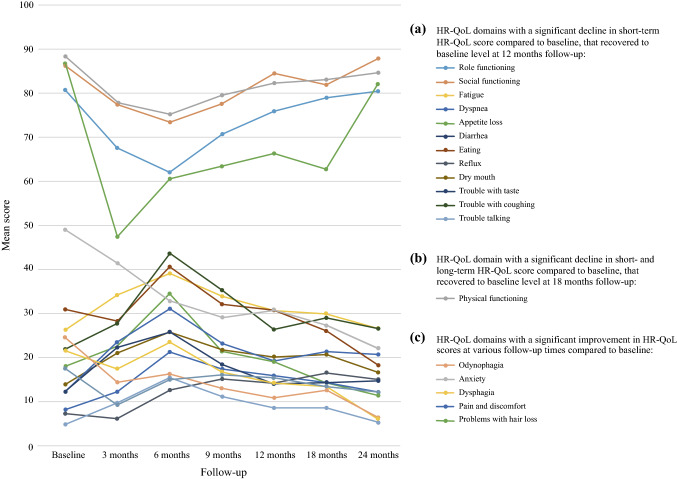
Change in health-related quality of life (HR-QoL) for patients with postoperative complications. **A** A significant decline in short-term HR-QoL score compared with baseline was found in the following 12 HR-QoL domains, which recovered to baseline level during the 12-month follow-up period: “role functioning” (at 3 and 6 months, *p *< 0.001; at 9 months, *p* = 0.027), “social functioning” (at 3 months, *p *= 0.010; at 6 months, *p *< 0.001; at 9 months, *p *= 0.035), “fatigue” (at 6 months, *p *< 0.001), “dyspnea” (at 3 and 6 months, *p *< 0.001; at 9 months, *p *= 0.006), “appetite loss” (at 6 months, *p *< 0.001), “diarrhea” (at 6 months, *p* < 0.001; at 9 months, *p *= 0.007), “eating” (at 6 months, *p *= 0.021), “reflux” (at 9 months, *p *= 0.013), “dry mouth” (at 6 months, *p *< 0.001), “trouble with taste” (at 6 months, *p *< 0.001), “trouble with coughing” (at 6 and 9 months, *p *< 0.001), and “trouble talking” (at 6 months, *p *< 0.001). **B** A significant decline in the “physical functioning” score was found during the follow-up visits at 3, 6, and 9 months (*p *< 0.001) and at 12 months (*p *= 0.045), which had recovered to baseline level at the 18-month follow-up visit. **C** In the following five HR-QoL domains, an improved HR-QoL score compared with baseline was found at all the follow-up times: “odynophagia” (at 3 months, *p* = 0.001; at 6 months, *p *= 0.029; at 9, 12, 18, and 24 months, *p *< 0.001), “anxiety” (at 9, 12, 18, and 24 months, *p *< 0.001), “pain and discomfort” (at 3 months, *p *= 0.006), “problems with hair loss,” *p *< 0.001), and“dysphagia” (at 24 months, *p *< 0.001).

A univariable linear regression analysis of the HR-QoL domains was performed between baseline and the 3-, 6-, 9-, 12-, 18-, and 24-month follow-up evaluations for the patients with postoperative complications (Table S4). In 10 HR-QoL domains (“role functioning” and “dyspnea” [at 3, 6, and 9 months], “trouble with coughing” [at 6 and 9 months], “social functioning,” “fatigue,” “appetite loss,” “dry mouth,” “trouble with taste,” “diarrhea,” and “trouble talking” [at 6 months]), scores denoting clinically relevant and significantly more impaired short-term HR-QoL compared with baseline were found, which had recovered to baseline level at the 12-month follow-up evaluation (Fig. 2).

### HR-QoL After Anastomotic Leakage

The HR-QoL scores were compared between the patients with anastomotic leakage (*n *= 83) and the patients without anastomotic leakage (*n *= 360) over time. After linear mixed-models analysis and Bonferroni correction, no *p* value below 0.001 was found in any of the domains (data not shown). The HR-QoL scores also were compared between the patients with severe (grade 2 or 3) anastomotic leakage (*n *= 54) and the patients with grade 1 or no anastomotic leakage (*n* = 432) over time (Table S5). After linear mixed-models analysis and Bonferroni correction, a significant difference in HR-QoL over time was found in the “choked when swallowing” domain (*p *< 0.001; Table 4). After univariable linear regression analysis and correction for multiple testing, the patients with grade 2 or 3 anastomotic leakage showed significantly more reported problems with “choking when swallowing” than the patients with grade 1 or no anastomotic leakage in follow-up evaluations at 6 months (mean difference, 14.5; 95% CI, − 24.833 to − 4.202; *p *= 0.049), 9 months (mean difference, 22.4; 95% CI, − 34.259 to − 10.591; *p *= 0.007), and 24 months (mean difference, 24.6; 95% CI, − 39.494 to − 9.727; *p *= 0.007) (Table 5). The mean scores differed more than 10 points and were therefore clinically relevant.

**Table 4:** Linear mixed-models analysis of health-related quality of life (HR-QoL) scores at baseline and at the 3-, 6-, 9-, 12-, 18-, and 24-months follow-up visits for patients with grade 1 or no anastomotic leakage (−) and patients with grade 2 or 3 anastomotic leakage (+) after esophagectomy

	Mean HR-QoL score^A^							
	Baseline	3 months	6 months	9 months	12 months	18 months	24 months	Corrected *p* value^B^
*N* (−)	432	358	342	302	271	189	107	
*N* (+)	54	40	36	29	25	15	6	
EORTC QLQ-C30								
Global health								
** −**	74.3	71.0	69.2	73.2	73.6	73.7	75.7	3.007
** +**	74.9	68.6	65.2	69.6	69.8	69.5	67.3	
Functioning scores								
Physical functioning								
−	88.4	79.5	77.2	81.1	81.2	82.2	82.7	0.009
** +**	88.4	67.5	65.1	72.4	75.3	75.0	68.4	
Role functioning								
** −**	81.1	67.6	65.2	72.6	75.5	76.5	75.6	0.155
** +**	81.2	63.2	43.9	62.2	69.1	75.6	52.4	
Emotional functioning								
** −**	78.9	81.9	84.4	84.2	84.7	83.4	85.0	3.162
** +**	74.8	80.6	78.8	78.2	78.4	81.2	81.7	
Cognitive functioning								
** −**	89.0	86.5	85.8	85.1	85.8	85.1	84.6	11.780
** +**	89.8	80.8	81.3	81.9	84.0	86.6	82.0	
Social functioning								
** −**	84.9	76.9	75.1	79.8	84.3	82.7	85.2	0.806
** +**	84.6	72.8	65.0	67.6	79.0	74.4	80.7	
Symptom scores								
Fatigue								
** −**	25.5	34.2	36.6	32.7	30.8	30.3	29.4	0.248
** +**	29.2	45.8	49.4	40.5	33.7	36.4	38.6	
Nausea and vomiting								
** −**	11.2	11.3	17.2	14.2	10.7	9.8	9.7	16.120
** +**	10.5	14.5	19.1	18.1	10.5	9.9	10.8	
Pain								
** −**	15.7	20.0	18.1	16.4	16.2	14.0	14.9	1.209
** +**	15.1	24.7	23.7	21.3	16.9	23.3	29.4	
Dyspnea								
** −**	11.6	20.0	26.2	20.0	18.9	19.0	16.6	0.008
** +**	13.6	31.8	40.6	32.3	28.5	30.3	38.2	
Insomnia								
** −**	23.4	26.7	22.8	19.7	22.0	21.6	20.7	14.508
** +**	24.1	32.6	28.5	18.8	21.8	24.5	24.3	
Appetite loss								
** −**	19.0	24.8	35.0	24.0	20.2	18.7	16.1	9.672
** +**	13.0	25.5	37.8	21.4	14.7	11.3	10.9	
Constipation								
** −**	14.1	13.5	11.0	11.1	9.7	8.7	11.3	24.025
** +**	11.1	18.1	15.1	8.0	8.7	12.0	11.2	
Diarrhea								
** −**	6.2	11.0	21.0	16.7	15.3	14.2	14.1	0.031
** +**	11.1	19.2	29.4	25.7	20.3	19.0	39.2	
Financial difficulties								
** −**	5.6	7.5	6.7	7.2	7.3	6.6	5.1	3.410
** +**	9.3	10.8	11.4	12.2	9.4	12.6	6.3	
EORTC QLQ-OG25								
Functioning								
Body image								
** −**	90.9	86.1	84.2	87.5	86.5	86.3	89.6	4.929
** +**	90.1	85.6	86.6	83.7	77.4	80.8	79.3	
Symptom scores								
Dysphagia								
** −**	22.1	18.1	19.8	14.7	13.7	11.5	9.4	2.635
** +**	17.0	23.0	33.8	24.6	18.3	13.8	5.6	
Eating								
** −**	32.2	29.0	38.8	31.1	28.9	26.1	24.6	3.968
** +**	25.1	36.6	48.0	40.5	33.5	32.9	27.0	
Reflux								
** −**	6.9	7.4	13.7	15.1	16.0	17.8	14.7	20.212
** +**	5.2	7.5	15.6	16.9	10.7	13.3	14.3	
Odynophagia								
** −**	24.5	15.4	15.0	12.1	10.8	9.5	7.5	6.727
** +**	21.6	15.8	22.2	17.1	12.2	14.5	8.5	
Pain and discomfort								
** −**	16.2	11.6	15.1	15.8	15.8	14.5	12.8	21.638
** +**	22.5	12.3	15.1	19.2	11.9	6.2	8.0	
Anxiety								
** −**	50.1	40.7	32.1	29.6	28.8	27.3	26.4	26.908
** +**	47.2	41.4	32.4	30.2	30.5	30.8	26.4	
Eating with others								
** −**	14.5	10.5	15.1	12.5	11.2	10.2	7.2	2.790
** +**	14.2	13.8	16.1	13.8	11.3	13.8	30.0	
Dry mouth								
** −**	13.1	19.0	25.6	20.9	20.1	20.3	21.8	25.048
** +**	14.2	27.0	25.4	19.2	16.2	11.7	21.2	
Trouble with taste								
** −**	11.7	20.0	24.2	17.1	16.5	14.1	14.7	25.327
** +**	6.2	31.8	24.6	18.1	12.0	9.4	21.2	
Trouble swallowing saliva								
** −**	9.1	13.9	15.8	12.5	13.5	13.7	10.4	4.929
** +**	9.9	14.4	13.4	16.3	22.6	19.2	20.7	
Choked when swallowing								
** −**	7.0	7.8	11.4	8.7	8.2	10.2	9.6	**<0.001**
** +**	9.9	12.6	26.5	28.5	20.7	14.3	21.6	
Trouble with coughing								
** −**	20.1	27.6	41.2	32.6	26.6	27.4	23.8	0.093
** +**	25.3	36.3	57.2	44.8	35.1	36.7	29.3	
Trouble talking								
** −**	4.6	9.9	13.6	8.1	7.6	6.6	4.0	6.169
** +**	4.3	11.0	18.9	13.0	12.8	16.7	− 4.8	
Weight loss								
** −**	17.9	18.2	23.5	22.0	19.9	16.3	16.9	7.843
** +**	19.5	22.0	22.0	29.7	24.1	16.8	27.0	
Problems with hair loss								
** −**	76.5	48.7	57.5	53.3	62.7	60.6	63.1	23.498
** +**	91.5	29.6	42.5	73.6	28.3	40.3	93.6	

*N* (−), number of patients with grade 1 or no anastomotic leakage; *N* (+), number of patients with grade 2–3 anastomotic leakage

^A^Values are represented as mean HR-QoL scores unless otherwise indicated.

^B^Corrected *p* value = corrected for multiple testing according to Bonferroni method. Bold *p* values represent significance (*p *< 0.001).

**Table 5:** Univariable linear regression analysis of health-related quality of life (HR-QoL) domain “choked when swallowing” over time for patients with grade 1 or no anastomotic leakage and patients with grade 2 or 3 anastomotic leakage after esophagectomy

Choked when swallowing	Univariable linear regression								
	Grade 1 or no anastomotic leakage		Grade 2 or 3 anastomotic leakage		B	95% CI		*p* Value	Corrected *p* value^A^
	Mean	SD	Mean	SD		Lower	Upper		
Baseline	7.0	17.7	9.9	20.1	− 2.9	− 7.993	2.226	0.268	1.876
At 3 months	7.9	16.2	12.3	26.2	− 4.4	− 13.176	4.345	0.315	2.205
At 6 months	11.4	19.1	25.9	29.9	− 14.5	− 24.833	–4.202	**0.007**	**0.049**
At 9 months	8.6	16.3	31.0	30.8	− 22.4	− 34.259	–10.591	**0.001**	**0.007**
At 12 months	7.8	16.1	20.8	21.6	− 13.0	− 22.308	–3.746	**0.008**	0.056
At 18 months	10.2	17.9	20.0	21.1	− 9.8	− 19.402	–0.168	0.046	0.322
At 24 months	8.7	16.1	33.3	23.6	− 24.6	− 39.494	–9.727	**0.001**	**0.007**

*SD* standard deviation; *B* regression coefficient; *CI* confidence interval

^A^Bold *p* values represent significance (*p *< 0.05). Corrected *p* value = corrected for multiple testing according to Bonferroni correction.

### HR-QoL After Cervical and Intrathoracic Anastomosis

The HR-QoL scores for the patients who had esophagectomy with a cervical anastomosis (*n *= 147) were compared over time with the HR-QoL scores of the patients who had esophagectomy with an intrathoracic anastomosis (*n *= 308). After linear mixed-models analysis and Bonferroni correction, no significant difference was found in any of the domains (data not shown).

## Discussion

This study investigated the difference in the short- and long-term HR-QoL for patients with and without postoperative complications after multimodality treatment including an esophagectomy with curative intent for esophageal or GEJ cancer in a nationwide cohort. The results of this study showed that in general, the short- and long-term HR-QoL does not differ between patients with and without postoperative complications after esophagectomy. However, grade 2 or 3 anastomotic leakage was found to affect “chocking when swallowing” compared with grade 1 or no anastomotic leakage.

The absence of differences in HR-QoL between the patients with and without postoperative complications is in contrast to our hypothesis. When investigated separately, in both groups a decline in various short-term HR-QoL domain scores was found that was restored to baseline level with time. The observed impairment in HR-QoL is therefore more likely to be attributable to functional complaints related to the reconstruction after esophagectomy, and remarkably, complications do not seem to influence this. A recent prospective multicenter study showed that the majority of patients have functional complaints that last up to more than 1 year after an esophagectomy.^29^ The authors found a relation between the absence of 30-day complications and HR-QoL, with an increased physical, social, role functioning, and global health status in the group without complications.

Few other studies have investigated the influence of postoperative complications on HR-QoL after an esophagectomy.^12–15^ Overall, an impaired HR-QoL was found at the 6-month follow-up evaluations of patients with postoperative complications versus patients without postoperative complications.^13–15^ Anastomotic leakage, one of the most severe postoperative complications associated with the development of strictures,^30^ was found to be associated with odynophagia and eating difficulties 6 months after an esophagectomy with an intrathoracic anastomosis.^15^ Only one study investigated the impact of major postoperative complications on long-term HR-QoL and found that the patients with major postoperative complications reported more dyspnea, fatigue, and eating restrictions 6 months, 3 years, and 5 years after the operation than the patients with no postoperative complications.^13^ The negative impact of postoperative complications on HR-QoL was found to last up to 10 years postoperatively.^12^ The majority of these studies reported only major postoperative complications, although no complication grading system was used to define the severity of the complications.^12–14^

In 2016, the national audit for upper GI cancer (DUCA) started with the registration of the Clavien-Dindo classification for postoperative complications. Between 2016 and 2017, the results showed that 1046 (65%) of 1617 patients had a complication after their esophagectomy. Altogether, 529 patients (33%) had pulmonary complications, with pneumonia as the most common complication (341 cases; 21% of all complications and 64% of all pulmonary complications). Of the patients with a complication, 29% had Clavien-Dindo grade 3 or higher complications.^1^ The majority of the patients in the current study may have had a complication below Clavien-Dindo grade 3, which may explain the absence of differences in HR-QoL found between the patients with and without postoperative complications. In addition, the patients with more severe anastomotic leakage (grade 2 or 3), reported more problems with “choking when swallowing” at the 6-, 9-, and 24-month follow-up evaluation. This accords with a previous study that found significantly more odynophagia and problems with eating among patients with anastomotic leakage than among patients without an anastomotic leakage 6 months after an esophagectomy with an intrathoracic anastomosis.^15^ In addition, because problems with eating also are known to be dependent on the anastomotic site,^31^ we performed an analysis comparing HR-QoL between the patients with cervical and intrathoracic anastomoses and found no significant difference in HR-QoL between these two groups. However, because the number of patients in the grades 2 and 3 anastomotic leakage group was limited, this finding may have been due to chance despite the use of a Bonferroni procedure.

This study had a number of limitations. Selection bias could have occurred because it was unknown how many patients were eligible and how many had died during the follow-up period. In addition, the reasons for declining participation were not recorded. The results also could have been influenced by the decline in the response rate in the long-term follow-up evaluation. Moreover, this was a population-based, non-randomized cohort study of patients who differed in the number of postoperative complications. Therefore, the two groups differed with respect to a number of baseline variables including occurrence of (pulmonary) comorbidities, surgical technique (open, minimally invasive, hybrid), surgical approach (transthoracic, transhiatal), and location of anastomosis (cervical, intrathoracic). No correction for confounders was performed because we aimed to investigate the difference in HR-QoL in a naturally occurring population considering age, gender, comorbidities, and surgical technique. Furthermore, recurrent laryngeal nerve palsy also likely is independently related to HR-QoL. However, only a small number of patients in this study (*n* = 13) had a recurrent laryngeal nerve palsy, so no reliable subgroup analysis could be performed. Also, it was not possible to investigate the influence of severity of complications according to Clavien-Dindo grade, nor to investigate the influence of separate pulmonary complications on HR-QoL because the NCR does not register these data.

A strength of this study was that it investigated a population-based prospective cohort, which counteracted the selection bias seen in randomized clinical trials that use strict inclusion criteria. Also, this study included a large sample of post-esophagectomy patients treated after implementation of improvements in esophageal cancer treatment, including minimally invasive surgery and neoadjuvant/perioperative therapy.

To counteract the bias of multiple testing, a Bonferroni correction was performed. Because the number of patients with grade 2 or 3 anastomotic leakage was relatively small, a more stringent *p* value (*p *< 0.001) was chosen for this subgroup analysis. Also, to prevent over-interpretation of the clinical relevance of the results, a mean HR-QoL score change of more than 10 points was considered clinically relevant for all the HR-QoL domains.

## Conclusion

Patients with and without complications after esophagectomy generally report comparable short- and long-term HR-QoL up to 24-months after surgery. In this study, both groups of patients showed a decline in short-term HR-QoL in various domains, which was restored to baseline levels with time. The patients with grade 2 or 3 anastomotic leakage reported worse HR-QoL in a single HR-QoL domain (“choking when swallowing”) than the patients with grade 1 or no anastomotic leakage. The temporary decrease in HR-QoL likely was related to the nature of the esophagectomy and reconstruction itself, and future research should focus on how to minimize these functional complaints.

## Supplementary Information

Below is the link to the electronic supplementary material.

Supplementary file1 (DOCX 168 kb)
