# Supplementary material for: Postoperative Complications and Long-Term Quality of Life After Multimodality Treatment for Esophageal Cancer: An Analysis of the Prospective Observational Cohort Study of Esophageal-Gastric Cancer Patients (POCOP)
Source: Ann Surg Oncol. 2021 May 25;28(12):7259–76. doi: 10.1245/s10434-021-10144-5 (PMC8519926; doi:10.1245/s10434-021-10144-5)
Supplement: Supplementary file 1 — Supplementary file1 (DOCX 168 kb) [file 10434_2021_10144_MOESM1_ESM.docx]

### Supplementary table 1: definitions of postoperative complications in The Netherlands Cancer Registry.

| **Anastomotic leakage**: full thickness defect of the esophagus, anastomosis, staple line or tube of the stomach regardless of the method of identification. Register anastomotic leakage regardless of grade. | Grading:  I: Leakage for which conservative policy is used such as: expectant, medicinal or nutritional measure.  II: Leakage requiring non-surgical intervention such as drain, stent or neck wound opening in ward.  III: Leakage requiring surgical intervention.  **Anastomotic leakage coding:**  0 none  1 anastomotic leak, grade I.  2 anastomotic leakage, grade II  3 anastomotic leakage, grade III  6 anastomotic leakage, grade unknown  8 not applicable  9 unknown |
| --- | --- |
| **Pulmonary complication**, which includes: | • Pneumonia  • Pleural effusion (accumulation of an abnormal amount of fluid in the pleural space) requiring drainage  • Pneumothorax (collapsed lung) that requires treatment  • Atelectasis (loss of lung volume) requiring bronchoscopy  • Respiratory failure requiring re-intubation and ventilation  • Acute aspiration  • Tracheobronchial injury (TBI) = broncho-esophageal fistula  • ARDS (Acute Respiratory Distress Syndrome) fluid accumulation in the lungs (pulmonary edema) and low oxygen levels in the blood.  • Prolonged chest drainage due to air leakage> 10 days |
| **Cardiac complication**, which includes: | • Cardiac arrest requiring resuscitation  • (Acute) Myocardial infarction = heart infarction. AMI = acute myocardial infarction.  • Atrial fibrillation, atrial dysrhythmia, atrial flutter, requiring treatment  • Ventricular dysrhythmia (chamber / ventricular fibrillation (VF)) requiring treatment  • Cardiac decompensation = Congestive heart failure (CHF), requiring treatment  • Pericardial fluid requiring treatment |
| **Thromboembolic complications**, including: | • Deep vein thrombosis (DVT)  • Pulmonary embolism  • Thrombophlebitis / superficial venous thrombosis (thrombotic process in a superficial vein with signs of inflammation). |
| **Chyle** **leakage**: Chyle leakage may develop postoperatively as a result of damage to the lymph vessels during surgery. Record chyle leakage regardless of grade. | Grading:  I: Chyle leakage requiring MCT diet (low fat diet)  II: Chyle leakage for which TPV (total parenteral nutrition) is required  III: Chyle leakage requiring intervention |
| **Recurrent nerve injury** = inferior laryngic nerve = recurrent laryngeal nerve = laryngeal nerve. Register recurrent nerve injuries regardless of grade. | Grading:  I: Temporary outage for which no treatment  II: Permanent failure requiring elective surgical intervention  III: Permanent failure requiring acute surgical intervention |
| **Neurological complication other than recurrent nerve injury**, including: | • TIA (Transient Ischaemic Attack), transient stroke.  • CVA = Cerebro Vascular Accident (stroke / seizure). Could be cerebral infarction or cerebral haemorrhage.  • Acute delirium = acute delirium = acute hallucinatory confusion with altered consciousness  • Delirium tremens (DTs)  • Other neurological injuries |
| **Wound abscess / infection**, which includes: | • Wound infection requiring wound opening or antibiotics  • Central line infection requiring removal or antibiotics  • Abscess (intrathoracic or intra-abdominal)  • Sepsis  • Other infections requiring antibiotics. |
| **General information:**  Register postoperative complications within 30 days of primary tumor resection.  Register one complication per group if multiple complications occur per main group.  Record anastomotic leakage within 30 days of primary tumor resection.  Register anastomotic leakage regardless of which anastomosis is leaking. So a leaking anastomosis that attaches part of the small intestine to another part of the small intestine in a Roux-Y reconstruction is also an anastomotic leakage. Places where anastomotic leakage may occur: gastroenterostomy (GE) anastomosis, duodenal stump, jejuno-jejunostomy, or entero-enterostomy. | |

### Supplementary table 2: Linear mixed models analysis of health-related quality of life scores at baseline and at 3, 6, 9, 12, 18 and 24 months follow-up including uncorrected p values in patients with (+) and without (-) postoperative complications following esophagectomy.

|  |  | **Mean HR-QoL score** | | | | | | |  |  |
| --- | --- | --- | --- | --- | --- | --- | --- | --- | --- | --- |
|  |  | **Baseline** | **3 months** | **6 months** | **9 months** | **12 months** | **18 months** | **24 months** | **p value** | **Corrected p value** |
|  | **N(+)** | **270** | **212** | **196** | **171** | **152** | **105** | **50** |  |  |
|  | **N(-)** | **216** | **187** | **182** | **162** | **145** | **99** | **63** |  |  |
| **EORTC QLQ-C30** | |  |  |  |  |  |  |  |  |  |
| **Global Health** | **+** | 74.5 | 71.4 | 68.3 | 72.6 | 72.0 | 71.9 | 73.5 | 0.343 | 10.633 |
|  | **-** | 74.3 | 70.2 | 69.2 | 73.1 | 74.6 | 74.7 | 76.6 |  |  |
| **Functioning scores** | |  |  |  |  |  |  |  |  |  |
| **Physical functioning** | **+** | 88.2 | 77.6 | 74.4 | 78.3 | 79.4 | 79.7 | 80.6 | 0.073 | 2.263 |
|  | **-** | 88.7 | 79.1 | 77.9 | 82.3 | 81.9 | 83.4 | 82.7 |  |  |
| **Role functioning** | **+** | 80.5 | 67.3 | 61.4 | 69.2 | 74.0 | 76.1 | 73.7 | 0.344 | 10.664 |
|  | **-** | 81.9 | 66.9 | 65.1 | 74.2 | 75.8 | 76.6 | 74.8 |  |  |
| **Emotional functioning** | **+** | 78.7 | 81.4 | 83.0 | 82.8 | 82.7 | 81.3 | 82.8 | 0.193 | 5.983 |
|  | **-** | 78.0 | 82.1 | 84.7 | 84.4 | 85.5 | 85.1 | 86.3 |  |  |
| **Cognitive functioning** | **+** | 89.3 | 85.7 | 84.5 | 85.9 | 84.7 | 84.4 | 83.5 | 0.687 | 21.297 |
|  | **-** | 89.0 | 86.1 | 86.2 | 83.5 | 86.5 | 85.9 | 85.2 |  |  |
| **Social functioning** | **+** | 86.1 | 77.1 | 73.0 | 76.7 | 83.2 | 80.5 | 85.3 | 0.664 | 20.584 |
|  | **-** | 83.3 | 75.8 | 75.2 | 80.7 | 84.3 | 83.4 | 84.4 |  |  |
| **Symptom scores** | |  |  |  |  |  |  |  |  |  |
| **Fatigue** | **+** | 26.2 | 34.1 | 39.4 | 35.1 | 32.6 | 31.9 | 31.0 | 0.333 | 10.323 |
|  | **-** | 25.5 | 36.8 | 36.1 | 31.7 | 29.6 | 29.8 | 29.3 |  |  |
| **Nausea and vomiting** | **+** | 11.0 | 10.3 | 17.6 | 16.1 | 12.5 | 9.9 | 9.6 | 0.599 | 18.569 |
|  | **-** | 11.2 | 13.1 | 17.2 | 12.9 | 8.8 | 9.8 | 9.7 |  |  |
| **Pain** | **+** | 16.4 | 20.5 | 19.7 | 17.8 | 17.1 | 16.1 | 18.0 | 0.174 | 5.394 |
|  | **-** | 14.7 | 20.4 | 17.4 | 15.8 | 15.4 | 13.2 | 13.7 |  |  |
| **Dyspnea** | **+** | 16.4 | 20.5 | 19.7 | 17.8 | 17.1 | 16.1 | 18.0 | **0.021** | 0.651 |
|  | **-** | 14.7 | 20.4 | 17.4 | 15.8 | 15.4 | 13.2 | 13.7 |  |  |
| **Insomnia** | **+** | 22.8 | 25.7 | 23.6 | 19.1 | 20.6 | 21.3 | 24.9 | 0.820 | 25.420 |
|  | **-** | 24.3 | 29.1 | 23.1 | 20.4 | 23.5 | 22.5 | 18.2 |  |  |
| **Appetite loss** | **+** | 18.0 | 22.8 | 34.6 | 22.8 | 19.7 | 15.7 | 12.4 | 0.135 | 4.185 |
|  | **-** | 18.7 | 27.0 | 36.0 | 24.9 | 19.8 | 20.7 | 18.6 |  |  |
| **Constipation** | **+** | 14.1 | 14.3 | 12.0 | 9.4 | 10.2 | 9.3 | 11.9 | 0.756 | 23.436 |
|  | **-** | 13.5 | 13.6 | 10.7 | 12.3 | 8.9 | 8.6 | 10.7 |  |  |
| **Diarrhea** | **+** | 8.2 | 12.2 | 21.5 | 17.4 | 15.4 | 14.7 | 12.7 | 0.753 | 23.343 |
|  | **-** | 4.9 | 11.4 | 22.2 | 17.7 | 16.2 | 14.6 | 18.3 |  |  |
| **Financial difficulties** | **+** | 5.8 | 7.6 | 8.2 | 8.1 | 7.9 | 8.3 | 6.8 | 0.436 | 13.516 |
|  | **-** | 6.3 | 8.2 | 6.2 | 7.4 | 7.3 | 6.1 | 4.1 |  |  |
| **EORTC QLQ-OG25** | |  |  |  |  |  |  |  |  |  |
| **Functioning scores** | |  |  |  |  |  |  |  |  |  |
| **Body image** | **+** | 90.5 | 85.1 | 85.7 | 86.8 | 84.8 | 84.2 | 88.3 | 0.499 | 15.469 |
|  | **-** | 91.2 | 87.2 | 83.1 | 87.6 | 86.7 | 87.7 | 89.6 |  |  |
| **Symptom scores** | |  |  |  |  |  |  |  |  |  |
| **Dysphagia** | **+** | 21.5 | 17.5 | 23.7 | 17.2 | 15.4 | 14.7 | 8.9 | 0.109 | 3.379 |
|  | **-** | 21.5 | 19.8 | 18.4 | 13.9 | 12.9 | 8.7 | 9.3 |  |  |
| **Eating** | **+** | 30.8 | 28.4 | 40.6 | 32.9 | 31.4 | 28.1 | 23.4 | 0.712 | 22.072 |
|  | **-** | 32.2 | 31.2 | 38.5 | 31.0 | 27.2 | 25.2 | 25.8 |  |  |
| **Reflux** | **+** | 7.2 | 6.4 | 12.8 | 14.6 | 14.8 | 16.1 | 13.4 | 0.259 | 8.029 |
|  | **-** | 6.0 | 8.6 | 14.9 | 16.0 | 16.5 | 19.0 | 15.7 |  |  |
| **Odynophagia** | **+** | 24.4 | 14.3 | 16.2 | 12.8 | 11.2 | 12.8 | 8.3 | 0.310 | 9.610 |
|  | **-** | 23.9 | 16.8 | 15.1 | 12.2 | 10.6 | 6.8 | 6.8 |  |  |
| **Pain and discomfort** | **+** | 17.4 | 9.5 | 15.0 | 16.1 | 15.3 | 13.7 | 14.0 | 0.778 | 24.118 |
|  | **-** | 16.4 | 14.1 | 15.3 | 16.2 | 15.9 | 14.3 | 11.5 |  |  |
| **Anxiety** | **+** | 48.8 | 41.2 | 32.5 | 30.0 | 31.4 | 29.1 | 28.3 | 0.395 | 12.245 |
|  | **-** | 50.9 | 40.4 | 31.7 | 29.2 | 26.5 | 26.0 | 24.7 |  |  |
| **Eating with others** | **+** | 14.0 | 10.4 | 16.9 | 12.5 | 10.8 | 11.1 | 10.7 | 0.612 | 18.972 |
|  | **-** | 15.0 | 11.3 | 13.3 | 12.7 | 11.7 | 9.9 | 6.8 |  |  |
| **Dry mouth** | **+** | 13.9 | 20.5 | 26.1 | 21.7 | 20.7 | 21.0 | 18.8 | 0.663 | 20.553 |
|  | **-** | 12.3 | 18.9 | 25.0 | 19.8 | 18.7 | 18.2 | 23.8 |  |  |
| **Trouble with taste** | **+** | 12.4 | 22.2 | 26.0 | 18.6 | 15.3 | 13.8 | 17.4 | 0.223 | 6.913 |
|  | **-** | 9.4 | 19.6 | 22.4 | 15.7 | 16.9 | 13.6 | 12.8 |  |  |
| **Trouble swallowing saliva** | **+** | 9.5 | 14.9 | 14.3 | 13.2 | 15.2 | 15.8 | 11.7 | 0.499 | 15.469 |
|  | **-** | 8.8 | 12.8 | 16.9 | 12.4 | 13.3 | 12.3 | 10.4 |  |  |
| **Choked when swallowing** | **+** | 7.7 | 8.0 | 14.3 | 12.8 | 11.6 | 12.9 | 12.1 | **0.024** | 0.744 |
|  | **-** | 6.8 | 8.6 | 11.5 | 8.1 | 7.1 | 8.4 | 8.8 |  |  |
| **Trouble with coughing** | **+** | 21.9 | 27.5 | 44.1 | 36.0 | 27.7 | 30.3 | 27.6 | 0.133 | 4.123 |
|  | **-** | 19.2 | 29.5 | 41.3 | 31.5 | 27.1 | 26.1 | 21.7 |  |  |
| **Trouble talking** | **+** | 4.8 | 9.7 | 15.5 | 10.9 | 9.4 | 8.8 | 4.9 | **0.045** | 1.395 |
|  | **-** | 4.3 | 10.4 | 12.6 | 6.1 | 6.7 | 5.9 | 2.3 |  |  |
| **Weight loss** | **+** | 17.6 | 17.7 | 22.7 | 24.7 | 20.6 | 16.6 | 19.9 | 0.679 | 21.049 |
|  | **-** | 18.7 | 19.6 | 24.2 | 20.6 | 19.9 | 16.2 | 15.4 |  |  |
| **Problems with hair loss** | **+** | 81.7 | 46.6 | 54.9 | 61.0 | 62.3 | 54.8 | 69.9 | 0.528 | 16.368 |
|  | **-** | 73.3 | 47.4 | 57.1 | 49.7 | 57.5 | 62.4 | 59.3 |  |  |

_N(+) = number of patients with postoperative complications, N(-) = number of patients without postoperative complications. Values are represented as mean HR-QoL scores. P value = p value over time. Corrected p value = p value over time that is corrected for multiple testing according to Bonferroni method. Bold p values represent significance (p<0.05)._

### Supplementary table 3: Univariable linear regression analysis of health-related quality of life scores of patients without postoperative complications following esophagectomy.

|  | | | | **Univariable analysis** | | | |  |
| --- | --- | --- | --- | --- | --- | --- | --- | --- |
|  | | **Mean score** | **Standard deviation** | **B** | **95% CI** | | **p value** | **Corrected p value** |
|  |  |  |  |  | **Lower** | **Upper** |  |  |
| **EORTC QLQ-C30** | |  |  |  |  |  |  |  |
| **Global Health** | **Baseline** | 74.3 | 17.8 |  |  |  |  |  |
|  | **3 months** | 70.4 | 19.3 | 4.0 | 0.313 | 7.604 | **0.033** | 6.138 |
|  | **6 months** | 69.4 | 15.8 | 5.0 | 1.637 | 8.285 | **0.004** | 0.744 |
|  | **9 months** | 72.9 | 17.3 | 1.4 | -2.188 | 5.017 | 0.441 | 82.026 |
|  | **12 months** | 74.9 | 17.4 | -0.6 | -4.343 | 3.148 | 0.754 | 140.244 |
|  | **18 months** | 76.3 | 14.9 | -1.9 | -5.715 | 1.878 | 0.32 | 9.920 |
|  | **24 months** | 77.9 | 15.5 | -3.6 | -8.455 | 1.323 | 0.152 | 28.272 |
| **Functioning scores** | |  |  |  |  |  |  |  |
| **Physical functioning** | **Baseline** | 88.7 | 14.6 |  |  |  |  |  |
|  | **3 months** | 79.6 | 18.7 | 9.1 | 5.804 | 12.472 | **<0.001** | **<0.001** |
|  | **6 months** | 78.2 | 16.7 | 10.5 | 7.365 | 13.618 | **<0.001** | **<0.001** |
|  | **9 months** | 82.0 | 16.9 | 6.7 | 3.506 | 9.922 | **<0.001** | **0.009** |
|  | **12 months** | 82.5 | 17.6 | 6.2 | 2.691 | 9.673 | **0.001** | 0.186 |
|  | **18 months** | 84.8 | 16.9 | 3.9 | 0.259 | 7.590 | **0.036** | 6.696 |
|  | **24 months** | 85.1 | 15.9 | 3.6 | -0.601 | 7.800 | 0.093 | 17.298 |
| **Role functioning** | **Baseline** | 81.9 | 25.4 |  |  |  |  |  |
|  | **3 months** | 67.1 | 32.9 | 14.7 | 8.890 | 20.597 | **<0.001** | **<0.001** |
|  | **6 months** | 65.9 | 28.1 | 15.9 | 10.646 | 21.215 | **<0.001** | **<0.001** |
|  | **9 months** | 73.8 | 27.1 | 8.1 | 2.698 | 13.434 | **0.003** | 0.558 |
|  | **12 months** | 76.0 | 26.6 | 5.8 | 0.338 | 11.299 | 0.038 | 7.068 |
|  | **18 months** | 77.9 | 24.4 | 3.9 | -2.073 | 9.902 | 0.199 | 37.014 |
|  | **24 months** | 78.0 | 24.8 | 3.8 | -3.300 | 10.936 | 0.292 | 54.312 |
| **Emotional functioning** | **Baseline** | 78.0 | 19.6 |  |  |  |  |  |
|  | **3 months** | 82.1 | 17.7 | -4.1 | -7.773 | -0.404 | **0.030** | 5.530 |
|  | **6 months** | 84.9 | 18.9 | -6.8 | -10.632 | -3.004 | **<0.001** | 0.092 |
|  | **9 months** | 84.1 | 18.4 | -6.1 | -10.015 | -2.204 | **0.002** | 0.372 |
|  | **12 months** | 85.9 | 18.1 | -7.9 | -11.919 | -3.886 | **<0.001** | **0.024** |
|  | **18 months** | 86.5 | 18.0 | -8.5 | -13.053 | -3.941 | **<0.001** | 0.053 |
|  | **24 months** | 88.5 | 15.0 | -10.5 | -15.030 | -5.884 | **<0.001** | **0.003** |
| **Cognitive functioning** | **Baseline** | 89.0 | 18.5 |  |  |  |  |  |
|  | **3 months** | 86.5 | 18.3 | 2.5 | -1.119 | 6.112 | 0.175 | 32.623 |
|  | **6 months** | 86.4 | 17.8 | 2.6 | -1.005 | 6.193 | 0.157 | 29.202 |
|  | **9 months** | 83.3 | 20.6 | 5.6 | 1.660 | 9.605 | **0.006** | 1.116 |
|  | **12 months** | 86.0 | 18.8 | 3.0 | -0.964 | 6.905 | 0.138 | 25.668 |
|  | **18 months** | 86.4 | 16.7 | 2.6 | -1.684 | 6.889 | 0.233 | 43.338 |
|  | **24 months** | 87.6 | 18.9 | 1.4 | -3.837 | 6.637 | 0.599 | 111.414 |
| **Social functioning** | **Baseline** | 83.3 | 23.6 |  |  |  |  |  |
|  | **3 months** | 76.0 | 25.8 | 7.3 | 2.503 | 12.192 | **0.003** | 0.566 |
|  | **6 months** | 75.9 | 23.4 | 7.5 | 2.799 | 12.118 | **0.002** | 0.372 |
|  | **9 months** | 81.4 | 23.7 | 2.0 | -2.874 | 6.808 | 0.425 | 79.05 |
|  | **12 months** | 85.0 | 21.5 | -1.6 | -6.447 | 3.206 | 0.510 | 94.86 |
|  | **18 months** | 85.2 | 21.8 | -1.9 | -7.361 | 3.658 | 0.509 | 94.674 |
|  | **24 months** | 87.0 | 19.5 | -3.7 | -10.121 | 2.714 | 0.257 | 47.802 |
| **Symptom scores** | |  |  |  |  |  |  |  |
| **Fatigue** | **Baseline** | 25.5 | 23.4 |  |  |  |  |  |
|  | **3 months** | 36.4 | 25.3 | -11.0 | -15.726 | -6.181 | **<0.001** | **0.002** |
|  | **6 months** | 35.6 | 21.6 | -10.1 | -14.596 | -5.671 | **<0.001** | **0.002** |
|  | **9 months** | 31.7 | 21.8 | -6.3 | -10.904 | -1.606 | **0.008** | 1.488 |
|  | **12 months** | 29.1 | 22.7 | -3.6 | -8.487 | 1.291 | 0.149 | 27.714 |
|  | **18 months** | 27.7 | 22.4 | -2.2 | -7.738 | 3.270 | 0.425 | 79.05 |
|  | **24 months** | 25.6 | 20.2 | -0.2 | -6.566 | 6.226 | 0.958 | 178.188 |
| **Nausea and vomiting** | **Baseline** | 11.2 | 19.1 |  |  |  |  |  |
|  | **3 months** | 13.2 | 19.7 | -2.0 | -5.828 | 1.822 | 0.304 | 56.530 |
|  | **6 months** | 17.1 | 20.8 | -5.9 | -9.835 | -1.939 | **0.004** | 0.744 |
|  | **9 months** | 12.9 | 18.8 | -1.7 | -5.559 | 2.253 | 0.406 | 75.516 |
|  | **12 months** | 9.3 | 13.5 | 2.0 | -1.402 | 5.364 | 0.250 | 46.500 |
|  | **18 months** | 10.3 | 15.2 | 1.0 | -3.323 | 5.265 | 0.657 | 122.202 |
|  | **24 months** | 10.1 | 15.4 | 1.2 | -3.986 | 6.360 | 0.652 | 121.272 |
| **Pain** | **Baseline** | 14.7 | 19.6 |  |  |  |  |  |
|  | **3 months** | 20.3 | 27.1 | -5.5 | -10.219 | -0.808 | **0.022** | 4.054 |
|  | **6 months** | 16.8 | 20.8 | -2.1 | -6.117 | 1.893 | 0.300 | 55.800 |
|  | **9 months** | 15.2 | 22.9 | -0.5 | -4.895 | 3.936 | 0.831 | 154.566 |
|  | **12 months** | 14.7 | 20.0 | 0.0 | -4.133 | 4.210 | 0.986 | 183.396 |
|  | **18 months** | 12.1 | 17.3 | 2.6 | -1.893 | 7.126 | 0.254 | 47.244 |
|  | **24 months** | 12.4 | 19.4 | 2.3 | -3.199 | 7.807 | 0.411 | 76.446 |
| **Dyspnea** | **Baseline** | 11.3 | 19.4 |  |  |  |  |  |
|  | **3 months** | 17.3 | 25.6 | -6.0 | -10.497 | -1.462 | **0.010** | 1.860 |
|  | **6 months** | 23.9 | 25.7 | -12.6 | -17.186 | -8.060 | **<0.001** | **<0.001** |
|  | **9 months** | 19.2 | 25.6 | -7.9 | -12.658 | -3.103 | **0.001** | 0.186 |
|  | **12 months** | 18.5 | 24.9 | -7.2 | -12.039 | -2.362 | **0.004** | 0.744 |
|  | **18 months** | 15.2 | 20.4 | -3.8 | -8.537 | 0.870 | 0.110 | 20.46 |
|  | **24 months** | 13.8 | 20.4 | -2.4 | -7.968 | 3.091 | 0.386 | 71.796 |
| **Insomnia** | **Baseline** | 13.5 | 22.3 |  |  |  |  |  |
|  | **3 months** | 13.2 | 23.8 | -4.8 | -10.837 | 1.140 | 0.112 | 20.832 |
|  | **6 months** | 23.0 | 27.7 | 1.3 | -4.322 | 6.963 | 0.646 | 120.156 |
|  | **9 months** | 20.3 | 27.0 | 4.0 | -1.794 | 9.805 | 0.175 | 32.550 |
|  | **12 months** | 22.5 | 28.7 | 1.9 | -4.229 | 8.004 | 0.544 | 101.184 |
|  | **18 months** | 20.5 | 27.6 | 3.8 | -3.034 | 10.638 | 0.275 | 51.150 |
|  | **24 months** | 15.9 | 22.3 | 8.5 | 1.671 | 15.265 | **0.015** | 2.790 |
| **Appetite loss** | **Baseline** | 24.3 | 29.0 |  |  |  |  |  |
|  | **3 months** | 29.2 | 31.9 | -8.2 | -13.973 | -2.441 | **0.005** | 0.930 |
|  | **6 months** | 35.4 | 33.1 | -16.8 | -22.700 | -10.832 | **<0.001** | **<0.001** |
|  | **9 months** | 24.9 | 29.3 | -6.3 | -12.050 | -0.636 | **0.029** | 5.394 |
|  | **12 months** | 19.7 | 26.9 | -1.1 | -6.586 | 4.443 | 0.7030 | 130.758 |
|  | **18 months** | 20.9 | 30.3 | -2.3 | -9.200 | 4.658 | 0.518 | 96.348 |
|  | **24 months** | 17.5 | 26.7 | 1.1 | -6.119 | 8.407 | 0.757 | 140.802 |
| **Constipation** | **Baseline** | 4.9 | 14.2 |  |  |  |  |  |
|  | **3 months** | 11.5 | 22.2 | 0.3 | -4.205 | 4.876 | 0.885 | 164.61 |
|  | **6 months** | 10.7 | 19.8 | 2.8 | -1.352 | 6.966 | 0.185 | 34.410 |
|  | **9 months** | 12.4 | 23.3 | 1.1 | -3.555 | 5.793 | 0.638 | 118.668 |
|  | **12 months** | 8.1 | 18.2 | 5.4 | 1.165 | 9.608 | **0.013** | 2.418 |
|  | **18 months** | 7.4 | 18.2 | 6.1 | 1.399 | 10.763 | **0.011** | 2.046 |
|  | **24 months** | 10.1 | 19.5 | 3.4 | -2.687 | 9.558 | 0.270 | 50.22 |
| **Diarrhea** | **Baseline** | 18.6 | 25.5 |  |  |  |  |  |
|  | **3 months** | 26.8 | 32.0 | -6.5 | -10.258 | -2.805 | **0.001** | 0.186 |
|  | **6 months** | 22.1 | 25.1 | -17.2 | -21.306 | -13.016 | **<0.001** | **<0.001** |
|  | **9 months** | 17.8 | 23.9 | -12.9 | -17.036 | -8.698 | **<0.001** | **<0.001** |
|  | **12 months** | 16.7 | 23.3 | -11.7 | -16.009 | -7.448 | **<0.001** | **<0.001** |
|  | **18 months** | 14.1 | 21.9 | -9.2 | -13.953 | -4.453 | **<0.001** | **<0.001** |
|  | **24 months** | 16.4 | 23.9 | -11.5 | -17.753 | -5.174 | **0.001** | 0.186 |
| **Financial difficulties** | **Baseline** | 6.3 | 17.2 |  |  |  |  |  |
|  | **3 months** | 7.9 | 20.5 | -1.6 | -5.301 | 2.099 | 0.396 | 73.656 |
|  | **6 months** | 5.2 | 16.4 | 1.2 | -2.167 | 4.508 | 0.491 | 91.326 |
|  | **9 months** | 6.2 | 17.2 | 0.1 | -3.402 | 3.633 | 0.948 | 176.328 |
|  | **12 months** | 6.7 | 17.9 | -0.4 | -4.083 | 3.311 | 0.838 | 155.868 |
|  | **18 months** | 6.5 | 16.3 | -0.1 | -4.194 | 3.923 | 0.948 | 176.328 |
|  | **24 months** | 3.7 | 12.1 | 2.6 | -1.183 | 6.430 | 0.175 | 32.550 |
| **EORTC QLQ-OG25** | |  |  |  |  |  |  |  |
| **Functioning scores** | |  |  |  |  |  |  |  |
| **Body image** | **Baseline** | 91.2 | 19.8 |  |  |  |  |  |
|  | **3 months** | 87.1 | 22.7 | 4.1 | -0.110 | 8.324 | 0.056 | 10.416 |
|  | **6 months** | 83.3 | 26.7 | 7.9 | 3.139 | 12.601 | **0.001** | 0.186 |
|  | **9 months** | 88.4 | 21.8 | 2.8 | -1.500 | 7.096 | 0.201 | 37.386 |
|  | **12 months** | 87.3 | 22.3 | 3.9 | -0.584 | 8.454 | 0.088 | 16.368 |
|  | **18 months** | 87.8 | 22.1 | 3.4 | -1.705 | 8.603 | 0.188 | 34.968 |
|  | **24 months** | 91.0 | 18.2 | 0.2 | -5.286 | 5.683 | 0.943 | 175.398 |
| **Symptom scores** | |  |  |  |  |  |  |  |
| **Dysphagia** | **Baseline** | 21.5 | 21.8 |  |  |  |  |  |
|  | **3 months** | 19.9 | 25.4 | 1.6 | -3.129 | 6.237 | 0.514 | 95.604 |
|  | **6 months** | 18.2 | 18.9 | 3.2 | -0.841 | 7.312 | 0.119 | 22.134 |
|  | **9 months** | 13.9 | 17.1 | 7.5 | 3.594 | 11.478 | **<0.001** | **0.037** |
|  | **12 months** | 12.8 | 17.0 | 8.7 | 4.633 | 12.702 | **<0.001** | **0.006** |
|  | **18 months** | 7.9 | 12.6 | 13.5 | 9.696 | 17.384 | **<0.001** | **<0.001** |
|  | **24 months** | 7.9 | 14.0 | 13.5 | 8.980 | 18.099 | **<0.001** | **<0.001** |
| **Eating** | **Baseline** | 32.2 | 26.9 |  |  |  |  |  |
|  | **3 months** | 31.1 | 30.5 | 1.1 | -4.647 | 6.749 | 0.717 | 133.362 |
|  | **6 months** | 38.6 | 24.9 | -6.5 | -11.631 | -1.288 | **0.014** | 2.604 |
|  | **9 months** | 31.5 | 24.4 | 0.7 | -4.612 | 5.972 | 0.801 | 148.986 |
|  | **12 months** | 26.8 | 23.2 | 5.4 | -0.014 | 10.765 | 0.051 | 9.486 |
|  | **18 months** | 24.4 | 21.7 | 7.8 | 1.683 | 13.864 | **0.013** | 2.418 |
|  | **24 months** | 22.2 | 21.9 | 9.9 | 2.637 | 17.219 | **0.008** | 1.488 |
| **Reflux** | **Baseline** | 12.3 | 22.5 |  |  |  |  |  |
|  | **3 months** | 19.0 | 28.5 | -2.6 | -5.826 | 0.565 | 0.106 | 19.716 |
|  | **6 months** | 15.0 | 22.3 | -9.0 | -12.751 | -5.212 | **<0.001** | **0.001** |
|  | **9 months** | 16.9 | 24.2 | -10.9 | -15.048 | -6.662 | **<0.001** | **<0.001** |
|  | **12 months** | 17.4 | 24.2 | -11.3 | -15.742 | -6.944 | **<0.001** | **<0.001** |
|  | **18 months** | 19.7 | 26.0 | -13.7 | -19.246 | -8.172 | **<0.001** | **0.001** |
|  | **24 months** | 15.6 | 23.5 | -9.6 | -15.797 | -3.383 | **0.003** | 0.558 |
| **Odynophagia** | **Baseline** | 16.4 | 22.4 |  |  |  |  |  |
|  | **3 months** | 14.1 | 22.4 | 7.2 | 2.313 | 12.012 | **0.004** | 0.744 |
|  | **6 months** | 14.8 | 18.3 | 9.1 | 4.872 | 13.358 | **<0.001** | **0.006** |
|  | **9 months** | 12.2 | 19.0 | 11.7 | 7.294 | 16.115 | **<0.001** | **<0.001** |
|  | **12 months** | 11.1 | 15.6 | 12.8 | 8.640 | 16.978 | **<0.001** | **<0.001** |
|  | **18 months** | 6.6 | 10.6 | 17.3 | 13.378 | 21.196 | **<0.001** | **<0.001** |
|  | **24 months** | 6.3 | 11.4 | 17.6 | 13.233 | 21.908 | **<0.001** | **<0.001** |
| **Pain and discomfort** | **Baseline** | 50.9 | 26.0 |  |  |  |  |  |
|  | **3 months** | 40.1 | 25.7 | 2.2 | -2.200 | 6.628 | 0.325 | 60.45 |
|  | **6 months** | 15.3 | 22.6 | 1.1 | -3.383 | 5.543 | 0.634 | 117.924 |
|  | **9 months** | 15.9 | 22.8 | 0.4 | -4.208 | 5.040 | 0.86 | 159.96 |
|  | **12 months** | 15.7 | 22.0 | 0.6 | -4.088 | 5.323 | 0.797 | 148.242 |
|  | **18 months** | 13.6 | 20.4 | 2.8 | -2.466 | 7.971 | 0.3 | 55.8 |
|  | **24 months** | 10.6 | 17.8 | 5.8 | 0.414 | 11.138 | **0.035** | 6.51 |
| **Anxiety** | **Baseline** | 15.0 | 26.5 |  |  |  |  |  |
|  | **3 months** | 11.5 | 23.4 | 10.9 | 5.794 | 15.950 | **<0.001** | **0.006** |
|  | **6 months** | 31.6 | 25.4 | 19.4 | 14.254 | 24.450 | **<0.001** | **<0.001** |
|  | **9 months** | 30.0 | 26.1 | 20.9 | 15.575 | 26.235 | **<0.001** | **<0.001** |
|  | **12 months** | 26.9 | 24.8 | 24.1 | 18.681 | 29.468 | **<0.001** | **<0.001** |
|  | **18 months** | 25.3 | 23.1 | 25.6 | 19.570 | 31.602 | **<0.001** | **<0.001** |
|  | **24 months** | 23.8 | 22.7 | 27.1 | 19.994 | 34.239 | **<0.001** | **<0.001** |
| **Eating with others** | **Baseline** | 6.0 | 14.0 |  |  |  |  |  |
|  | **3 months** | 8.6 | 17.9 | 3.5 | -1.409 | 8.397 | 0.162 | 30.132 |
|  | **6 months** | 12.8 | 23.8 | 2.1 | -2.901 | 7.141 | 0.407 | 75.702 |
|  | **9 months** | 12.1 | 21.3 | 2.9 | -1.962 | 7.734 | 0.243 | 45.198 |
|  | **12 months** | 11.7 | 23.2 | 3.3 | -1.887 | 8.515 | 0.211 | 39.246 |
|  | **18 months** | 9.2 | 19.0 | 5.8 | 0.601 | 10.970 | **0.029** | 5.394 |
|  | **24 months** | 5.8 | 15.3 | 9.1 | 3.943 | 14.356 | **0.001** | 0.186 |
| **Dry mouth** | **Baseline** | 9.4 | 18.7 |  |  |  |  |  |
|  | **3 months**  **3 months** | 19.7 | 28.0 | -6.7 | -11.754 | -1.548 | **0.011** | 2.046 |
|  | **6 months** | 24.4 | 31.2 | -12.1 | -17.585 | -6.613 | **<0.001** | **0.004** |
|  | **9 months** | 19.9 | 27.1 | -7.6 | -12.763 | -2.378 | **0.004** | 0.744 |
|  | **12 months** | 18.1 | 25.8 | -5.7 | -10.911 | -0.509 | **0.032** | 5.952 |
|  | **18 months** | 18.7 | 27.1 | -6.4 | -12.560 | -0.164 | **0.044** | 8.184 |
|  | **24 months** | 21.2 | 27.0 | -8.8 | -16.228 | -1.409 | **0.001** | 0.186 |
| **Trouble with taste** | **Baseline** | 8.8 | 19.8 |  |  |  |  |  |
|  | **3 months** | 12.9 | 22.7 | -10.3 | -15.058 | -5.541 | **<0.001** | **0.005** |
|  | **6 months** | 22.4 | 29.2 | -13.0 | -17.961 | -8.027 | **<0.001** | **<0.001** |
|  | **9 months** | 15.3 | 24.7 | -5.9 | -10.488 | -1.326 | **0.012** | 2.232 |
|  | **12 months** | 16.2 | 25.8 | -6.8 | -11.719 | -1.861 | **0.007** | 1.302 |
|  | **18 months** | 13.6 | 21.8 | -4.2 | -9.223 | 0.839 | 0.102 | 18.972 |
|  | **24 months** | 10.6 | 18.8 | -1.2 | -6.447 | 4.110 | 0.663 | 123.318 |
| **Trouble swallowing saliva** | **Baseline** | 6.8 | 18.1 |  |  |  |  |  |
|  | **3 months** | 8.6 | 16.6 | -4.1 | -8.324 | 0.110 | 0.056 | 10.416 |
|  | **6 months** | 16.7 | 26.7 | -7.9 | -12.601 | -3.139 | **0.001** | 0.186 |
|  | **9 months** | 11.6 | 21.8 | -2.8 | -7.096 | 1.500 | 0.201 | 37.386 |
|  | **12 months** | 12.7 | 22.3 | -3.9 | -8.454 | 0.584 | 0.088 | 16.368 |
|  | **18 months** | 12.2 | 22.1 | -3.4 | -8.603 | 1.706 | 0.188 | 34.968 |
|  | **24 months** | 9.0 | 18.2 | -0.2 | -5.683 | 5.286 | 0.943 | 175.398 |
| **Choked when swallowing** | **Baseline** | 19.2 | 21.7 |  |  |  |  |  |
|  | **3 months** | 28.8 | 29.1 | -1.8 | -5.259 | 1.605 | 0.296 | 55.056 |
|  | **6 months** | 11.3 | 18.0 | -4.5 | -8.060 | -0.889 | **0.015** | 2.790 |
|  | **9 months** | 8.1 | 14.8 | -1.3 | -4.688 | 2.182 | 0.474 | 88.164 |
|  | **12 months** | 7.4 | 15.0 | -0.6 | -4.167 | 2.996 | 0.748 | 139.128 |
|  | **18 months** | 8.9 | 15.6 | -2.1 | -6.290 | 2.064 | 0.320 | 59.520 |
|  | **24 months** | 7.9 | 14.3 | -1.1 | -5.996 | 3.767 | 0.653 | 121.458 |
| **Trouble with coughing** | **Baseline** | 4.3 | 14.1 |  |  |  |  |  |
|  | **3 months** | 10.3 | 20.5 | -9.6 | -14.778 | -4.513 | **<0.001** | **0.047** |
|  | **6 months** | 40.8 | 30.5 | -21.6 | -26.975 | -16.271 | **<0.001** | **<0.001** |
|  | **9 months** | 32.1 | 28.0 | -12.9 | -17.981 | -7.852 | **<0.001** | **<0.001** |
|  | **12 months** | 27.5 | 24.7 | -8.4 | -13.255 | -3.520 | **0.001** | 0.186 |
|  | **18 months** | 27.2 | 24.6 | -8.1 | -13.485 | -2.619 | **0.0040** | 0.744 |
|  | **24 months** | 21.7 | 24.8 | -2.5 | -8.866 | 3.798 | 0.431 | 80.166 |
| **Trouble talking** | **Baseline** | 18.7 | 25.6 |  |  |  |  |  |
|  | **3 months** | 19.5 | 26.8 | -6.0 | -9.528 | -2.482 | **0.001** | 0.186 |
|  | **6 months** | 12.6 | 22.6 | -8.3 | -12.086 | -4.457 | **<0.001** | **0.005** |
|  | **9 months** | 6.0 | 14.4 | -1.7 | -4.602 | 1.236 | 0.258 | 47.988 |
|  | **12 months** | 6.3 | 15.3 | -2.0 | -5.176 | 1.142 | 0.210 | 39.060 |
|  | **18 months** | 4.8 | 13.6 | -0.5 | -3.841 | 2.861 | 0.774 | 143.964 |
|  | **24 months** | 2.1 | 8.2 | 2.2 | -0.575 | 4.984 | 0.119 | 22.134 |
| **Weight loss** | **Baseline** | 76.9 | 65.6 |  |  |  |  |  |
|  | **3 months** | 48.7 | 54.2 | -0.8 | -5.943 | 4.370 | 0.764 | 142.104 |
|  | **6 months** | 23.7 | 27.6 | -5.0 | -10.301 | 0.239 | 0.061 | 11.346 |
|  | **9 months** | 20.1 | 25.6 | -1.4 | -6.658 | 3.838 | 0.598 | 111.228 |
|  | **12 months** | 18.6 | 25.5 | 0.0 | -5.402 | 5.451 | 0.993 | 184.698 |
|  | **18 months** | 15.3 | 26.3 | 3.4 | -2.827 | 9.560 | 0.286 | 53.196 |
|  | **24 months** | 13.2 | 20.3 | 5.4 | -0.690 | 11.580 | 0.081 | 15.066 |
| **Problems with hair loss** | **Baseline** | 23.9 | 24.6 |  |  |  |  |  |
|  | **3 months** | 16.8 | 24.6 | 28.2 | 11.500 | 44.814 | **0.001** | 0.186 |
|  | **6 months** | 58.2 | 58.2 | 18.7 | 1.322 | 36.082 | **0.035** | 6.510 |
|  | **9 months** | 60.0 | 60.6 | 16.9 | -2.047 | 35.834 | 0.08 | 14.880 |
|  | **12 months** | 64.1 | 62.4 | 12.8 | -6.859 | 32.376 | 0.201 | 37.386 |
|  | **18 months** | 70.6 | 63.5 | 6.3 | -16.251 | 28.862 | 0.581 | 108.066 |
|  | **24 months** | 66.7 | 64.5 | 10.2 | -16.463 | 36.917 | 0.449 | 83.514 |

_B = regression coefficient. 95% CI = confidence interval. Bold p values represent significance (p<0.05). Corrected p value = corrected according to the Bonferroni correction for multiple testing._

### Supplementary table 4: Univariable linear regression analysis of health-related quality of life scores of patients with postoperative complications following esophagectomy.

|  | |  |  | **Univariable analysis** | | | |  |
| --- | --- | --- | --- | --- | --- | --- | --- | --- |
|  |  | **Mean score** | **Standard deviation** | **B** | **95% CI** | | **p value** | **Corrected p value** |
|  |  |  |  |  | **Lower** | **Upper** |  |  |
| **EORTC QLQ-C30** | |  |  |  |  |  |  |  |
| **Global Health** | **Baseline** | 74.4 | 17.3 |  |  |  |  |  |
|  | **3 months** | 71.8 | 19.3 | 2.6 | -0.667 | 5.940 | 0.117 | 21.762 |
|  | **6 months** | 68.7 | 17.4 | 5.7 | 2.504 | 8.939 | **0.001** | 0.186 |
|  | **9 months** | 73.5 | 17.7 | 0.9 | -2.490 | 4.245 | 0.609 | 113.274 |
|  | **12 months** | 73.8 | 16.3 | 0.6 | -2.824 | 3.940 | 0.746 | 138.756 |
|  | **18 months** | 73.6 | 17.0 | 0.8 | -3.066 | 4.737 | 0.674 | 125.364 |
|  | **24 months** | 77.2 | 16.9 | -2.8 | -7.985 | 2.465 | 0.300 | 55.800 |
| **Functioning scores** | |  |  |  |  |  |  |  |
| **Physical functioning** | **Baseline** | 88.2 | 15.3 |  |  |  |  |  |
|  | **3 months** | 77.8 | 22.5 | 10.4 | 6.860 | 13.957 | **<0.001** | **<0.001** |
|  | **6 months** | 75.0 | 20.4 | 13.2 | 9.796 | 16.586 | **<0.001** | **<0.001** |
|  | **9 months** | 79.4 | 19.5 | 8.8 | 5.296 | 12.224 | **<0.001** | **<0.001** |
|  | **12 months** | 82.2 | 17.3 | 6.0 | 2.824 | 9.217 | **<0.001** | **0.045** |
|  | **18 months** | 82.9 | 16.1 | 5.3 | 1.744 | 8.765 | **0.003** | 0.558 |
|  | **24 months** | 84.6 | 15.9 | 3.6 | -1.069 | 8.252 | 0.131 | 24.366 |
| **Role functioning** | **Baseline** | 80.5 | 25.7 |  |  |  |  |  |
|  | **3 months** | 67.5 | 33.1 | 13.0 | 7.612 | 18.470 | **<0.001** | **<0.001** |
|  | **6 months** | 61.9 | 29.9 | 18.6 | 13.512 | 23.666 | **<0.001** | **<0.001** |
|  | **9 months** | 70.5 | 28.0 | 10.0 | 4.879 | 15.128 | **<0.001** | **0.027** |
|  | **12 months** | 75.7 | 26.4 | 4.8 | -0.341 | 10.013 | 0.067 | 12.462 |
|  | **18 months** | 78.7 | 23.3 | 1.8 | -3.903 | 7.431 | 0.541 | 100.626 |
|  | **24 months** | 80.3 | 23.5 | 0.2 | -7.528 | 7.849 | 0.967 | 179.862 |
| **Emotional functioning** | **Baseline** | 78.7 | 20.1 |  |  |  |  |  |
|  | **3 months** | 81.1 | 20.3 | -2.5 | -6.117 | 1.180 | 0.185 | 34.41 |
|  | **6 months** | 83.1 | 19.4 | -4.4 | -8.083 | -0.757 | **0.018** | 3.348 |
|  | **9 months** | 83.3 | 20.2 | -4.6 | -8.472 | -0.720 | **0.020** | 3.720 |
|  | **12 months** | 83.7 | 18.9 | -5.1 | -8.982 | -1.141 | **0.012** | 2.232 |
|  | **18 months** | 82.9 | 19.7 | -4.3 | -8.800 | 0.238 | 0.063 | 11.718 |
|  | **24 months** | 87.3 | 17.4 | -8.7 | -14.642 | -2.713 | **0.004** | 0.744 |
| **Cognitive functioning** | **Baseline** | 89.2 | 17.6 |  |  |  |  |  |
|  | **3 months** | 85.5 | 21.3 | 3.7 | 0.164 | 7.323 | **0.040** | 7.440 |
|  | **6 months** | 84.5 | 20.4 | 4.8 | 1.286 | 8.252 | **0.007** | 1.302 |
|  | **9 months** | 86.0 | 17.4 | 3.2 | -0.139 | 6.617 | 0.060 | 11.160 |
|  | **12 months** | 85.5 | 20.2 | 3.7 | -0.013 | 7.399 | 0.051 | 9.486 |
|  | **18 months** | 85.7 | 18.8 | 3.5 | -0.560 | 7.570 | 0.091 | 16.926 |
|  | **24 months** | 87.3 | 18.9 | 1.9 | -3.515 | 7.287 | 0.493 | 91.698 |
| **Social functioning** | **Baseline** | 86.1 | 19.8 |  |  |  |  |  |
|  | **3 months** | 77.3 | 25.7 | 8.8 | 4.534 | 12.982 | **<0.001** | **0.010** |
|  | **6 months** | 73.2 | 24.9 | 12.9 | 8.615 | 17.112 | **<0.001** | **<0.001** |
|  | **9 months** | 77.5 | 24.7 | 8.5 | 4.083 | 12.938 | **<0.001** | **0.035** |
|  | **12 months** | 84.3 | 21.4 | 1.7 | -2.328 | 5.807 | 0.401 | 74.586 |
|  | **18 months** | 81.7 | 24.5 | 4.3 | -0.980 | 9.607 | 0.110 | 20.460 |
|  | **24 months** | 87.7 | 18.7 | -1.6 | -7.560 | 4.345 | 0.596 | 110.856 |
| **Symptom scores** | |  |  |  |  |  |  |  |
| **Fatigue** | **Baseline** | 26.2 | 21.6 |  |  |  |  |  |
|  | **3 months** | 34.1 | 26.8 | -7.9 | -12.325 | -3.441 | **0.001** | 0.186 |
|  | **6 months** | 39.0 | 23.1 | -12.8 | -16.922 | -8.717 | **<0.001** | **<0.001** |
|  | **9 months** | 33.8 | 22.4 | -7.6 | -11.827 | -3.393 | **<0.001** | 0.080 |
|  | **12 months** | 30.5 | 22.5 | -4.3 | -8.709 | 0.027 | 0.051 | 9.486 |
|  | **18 months** | 29.9 | 21.3 | -3.7 | -8.540 | 1.179 | 0.137 | 25.482 |
|  | **24 months** | 26.4 | 22.7 | -0.2 | -6.823 | 6.353 | 0.944 | 175.584 |
| **Nausea and vomiting** | **Baseline** | 11.0 | 18.1 |  |  |  |  |  |
|  | **3 months** | 10.2 | 18.5 | 0.8 | -2.497 | 4.119 | 0.630 | 117.18 |
|  | **6 months** | 17.4 | 20.2 | -6.3 | -9.811 | -2.791 | **<0.001** | 0.086 |
|  | **9 months** | 15.5 | 20.6 | -4.4 | -8.117 | -0.765 | **0.018** | 3.348 |
|  | **12 months** | 12.0 | 18.3 | -0.9 | -4.531 | 2.726 | 0.625 | 116.250 |
|  | **18 months** | 9.2 | 12.9 | 1.8 | -1.448 | 5.134 | 0.271 | 50.406 |
|  | **24 months** | 8.3 | 14.4 | 2.7 | -2.617 | 8.049 | 0.317 | 58.962 |
| **Pain** | **Baseline** | 16.4 | 20.7 |  |  |  |  |  |
|  | **3 months** | 20.8 | 25.2 | -4.4 | -8.605 | -0.188 | **0.041** | 7.626 |
|  | **6 months** | 19.5 | 22.9 | -3.1 | -7.175 | 0.945 | 0.132 | 24.607 |
|  | **9 months** | 17.0 | 20.7 | -0.6 | -4.593 | 3.387 | 0.767 | 142.662 |
|  | **12 months** | 15.7 | 22.7 | 0.7 | -3.602 | 4.959 | 0.756 | 140.616 |
|  | **18 months** | 14.3 | 20.7 | 2.1 | -2.619 | 6.763 | 0.271 | 50.406 |
|  | **24 months** | 13.7 | 19.0 | 2.7 | -3.513 | 8.896 | 0.394 | 73.284 |
| **Dyspnea** | **Baseline** | 12.2 | 20.2 |  |  |  |  |  |
|  | **3 months** | 23.4 | 27.8 | -11.2 | -15.632 | -6.685 | **<0.001** | **<0.001** |
|  | **6 months** | 31.0 | 27.7 | -18.7 | -23.316 | -14.145 | **<0.001** | **<0.001** |
|  | **9 months** | 23.1 | 29.5 | -10.9 | -15.980 | -5.850 | **<0.001** | **0.006** |
|  | **12 months** | 19.3 | 27.3 | -7.1 | -12.060 | -2.092 | **0.006** | 1.116 |
|  | **18 months** | 21.3 | 24.1 | -9.0 | -14.283 | -3.812 | **0.001** | 0.186 |
|  | **24 months** | 20.7 | 27.7 | -8.4 | -16.653 | -0.236 | **0.044** | 8.184 |
| **Insomnia** | **Baseline** | 22.8 | 27.3 |  |  |  |  |  |
|  | **3 months** | 25.9 | 31.8 | -3.1 | -8.511 | 2.295 | 0.259 | 48.174 |
|  | **6 months** | 24.3 | 29.3 | -1.5 | -6.719 | 3.681 | 0.566 | 105.276 |
|  | **9 months** | 19.0 | 25.3 | 3.8 | -1.337 | 8.899 | 0.147 | 27.342 |
|  | **12 months** | 20.4 | 26.6 | 2.4 | -2.993 | 7.804 | 0.382 | 71.052 |
|  | **18 months** | 21.3 | 26.2 | 1.5 | -4.583 | 7.644 | 0.623 | 115.878 |
|  | **24 months** | 22.7 | 29.7 | 0.1 | -8.261 | 8.529 | 0.975 | 181.350 |
| **Appetite loss** | **Baseline** | 18.0 | 26.6 |  |  |  |  |  |
|  | **3 months** | 22.6 | 30.7 | -4.6 | -9.825 | 0.683 | 0.088 | 16.368 |
|  | **6 months** | 34.4 | 31.7 | -16.3 | -21.663 | -11.006 | **<0.001** | **<0.001** |
|  | **9 months** | 21.4 | 27.3 | -3.3 | -8.514 | 1.819 | 0.203 | 37.758 |
|  | **12 months** | 19.0 | 26.5 | -1.0 | -6.266 | 4.346 | 0.722 | 134.292 |
|  | **18 months** | 14.1 | 21.6 | 3.9 | -1.334 | 9.179 | 0.143 | 26.598 |
|  | **24 months** | 11.3 | 18.6 | 6.7 | 0.563 | 12.820 | **0.033** | 6.138 |
| **Constipation** | **Baseline** | 14.1 | 24.2 |  |  |  |  |  |
|  | **3 months** | 14.4 | 25.0 | -0.4 | -4.810 | 4.069 | 0.870 | 161.82 |
|  | **6 months** | 12.0 | 21.8 | 2.1 | -2.181 | 6.397 | 0.335 | 62.310 |
|  | **9 months** | 9.0 | 18.4 | 5.1 | 1.043 | 9.065 | **0.014** | 2.604 |
|  | **12 months** | 9.6 | 19.4 | 4.4 | 0.184 | 8.666 | **0.041** | 7.626 |
|  | **18 months** | 8.9 | 17.5 | 5.2 | 0.750 | 9.620 | **0.022** | 4.092 |
|  | **24 months** | 10.0 | 20.5 | 4.1 | -2.397 | 10.545 | 0.214 | 39.804 |
| **Diarrhea** | **Baseline** | 8.2 | 18.0 |  |  |  |  |  |
|  | **3 months** | 12.2 | 23.6 | -4.0 | -7.903 | -0.185 | **0.040** | 7.440 |
|  | **6 months** | 21.1 | 25.3 | -13.0 | -17.126 | -8.785 | **<0.001** | **<0.001** |
|  | **9 months** | 17.3 | 24.4 | -9.1 | -13.342 | -4.810 | **<0.001** | **0.007** |
|  | **12 months** | 15.8 | 23.3 | -7.6 | -11.915 | -3.307 | **0.001** | 0.186 |
|  | **18 months** | 14.3 | 23.0 | -6.1 | -11.049 | -1.166 | **0.016** | 2.976 |
|  | **24 months** | 12.0 | 23.1 | -3.8 | -10.711 | 3.068 | 0.272 | 50.592 |
| **Financial difficulties** | **Baseline** | 5.8 | 16.6 |  |  |  |  |  |
|  | **3 months** | 7.3 | 16.9 | -1.5 | -4.512 | 1.557 | 0.339 | 63.054 |
|  | **6 months** | 8.6 | 19.7 | -2.8 | -6.185 | 0.651 | 0.112 | 20.832 |
|  | **9 months** | 8.6 | 19.6 | -2.8 | -6.375 | 0.769 | 0.124 | 23.064 |
|  | **12 months** | 7.5 | 17.2 | -1.6 | -4.995 | 1.731 | 0.341 | 63.426 |
|  | **18 months** | 8.3 | 19.5 | -2.4 | -6.686 | 1.826 | 0.261 | 48.546 |
|  | **24 months** | 6.0 | 12.9 | -0.2 | -5.061 | 4.709 | 0.944 | 175.584 |
| **EORTC QLQ-OG25** | |  |  |  |  |  |  |  |
| **Functioning scores** | |  |  |  |  |  |  |  |
| **Body image** | **Baseline** | 90.5 | 19.9 |  |  |  |  |  |
|  | **3 months** | 85.3 | 24.0 | 5.2 | 1.145 | 9.225 | **0.012** | 2.232 |
|  | **6 months** | 86.1 | 23.4 | 4.4 | 0.351 | 8.507 | **0.033** | 6.138 |
|  | **9 months** | 87.5 | 22.0 | 3.1 | -1.035 | 7.156 | 0.142 | 26.412 |
|  | **12 months** | 85.8 | 26.0 | 4.7 | -0.084 | 9.553 | 0.054 | 10.044 |
|  | **18 months** | 87.0 | 24.7 | 3.5 | -1.799 | 8.855 | 0.193 | 35.898 |
|  | **24 months** | 90.0 | 18.1 | 0.5 | -5.429 | 6.453 | 0.866 | 161.076 |
| **Symptom scores** | |  |  |  |  |  |  |  |
| **Dysphagia** | **Baseline** | 21.5 | 22.4 |  |  |  |  |  |
|  | **3 months** | 17.4 | 24.5 | 4.1 | -0.104 | 8.368 | **0.056** | 10.416 |
|  | **6 months** | 23.5 | 24.7 | -2.0 | -6.338 | 2.313 | 0.361 | 67.146 |
|  | **9 months** | 16.7 | 20.8 | 4.8 | 0.620 | 9.021 | **0.025** | 4.65 |
|  | **12 months** | 14.2 | 18.2 | 7.3 | 3.122 | 11.515 | **0.001** | 0.186 |
|  | **18 months** | 13.3 | 19.7 | 8.2 | 3.334 | 13.145 | **0.001** | 0.186 |
|  | **24 months** | 6.0 | 11.5 | 15.5 | 11.320 | 19.720 | **<0.001** | **<0.001** |
| **Eating** | **Baseline** | 30.8 | 27.3 |  |  |  |  |  |
|  | **3 months** | 28.2 | 30.6 | 2.5 | -2.811 | 7.878 | 0.352 | 65.472 |
|  | **6 months** | 40.6 | 25.5 | -9.8 | -14.719 | -4.850 | **<0.001** | **0.021** |
|  | **9 months** | 32.0 | 24.9 | -1.3 | -6.346 | 3.842 | 0.629 | 116.994 |
|  | **12 months** | 30.7 | 24.7 | 0.1 | -5.057 | 5.236 | 0.973 | 180.978 |
|  | **18 months** | 26.1 | 23.2 | 4.7 | -1.257 | 10.668 | 0.122 | 22.692 |
|  | **24 months** | 18.2 | 20.7 | 12.6 | 5.857 | 19.246 | **<0.001** | 0.065 |
| **Reflux** | **Baseline** | 7.2 | 17.6 |  |  |  |  |  |
|  | **3 months** | 6.1 | 16.1 | 1.1 | -1.953 | 4.201 | 0.4730 | 87.978 |
|  | **6 months** | 12.5 | 19.9 | -5.3 | -8.849 | -1.809 | **0.003** | 0.558 |
|  | **9 months** | 15.1 | 21.2 | -7.9 | -11.725 | -4.043 | **<0.001** | **0.013** |
|  | **12 months** | 14.1 | 20.6 | -6.9 | -10.837 | -2.991 | **0.001** | 0.186 |
|  | **18 months** | 16.5 | 23.1 | -9.3 | -14.220 | -4.368 | **<0.001** | 0.051 |
|  | **24 months** | 15.0 | 18.5 | -7.8 | -13.438 | -2.134 | **0.008** | 1.488 |
| **Odynophagia** | **Baseline** | 24.5 | 25.7 |  |  |  |  |  |
|  | **3 months** | 14.3 | 22.5 | 10.2 | 5.792 | 14.529 | **<0.001** | **0.001** |
|  | **6 months** | 16.2 | 20.6 | 8.2 | 3.992 | 12.477 | **<0.001** | **0.029** |
|  | **9 months** | 13.0 | 17.0 | 11.5 | 7.456 | 15.490 | **<0.001** | **<0.001** |
|  | **12 months** | 10.8 | 17.2 | 13.7 | 9.514 | 17.792 | **<0.001** | **<0.001** |
|  | **18 months** | 12.7 | 18.4 | 11.8 | 7.105 | 16.513 | **<0.001** | **<0.001** |
|  | **24 months** | 6.3 | 12.1 | 18.1 | 13.549 | 22.724 | **<0.001** | **<0.001** |
| **Pain and discomfort** | **Baseline** | 17.4 | 23.9 |  |  |  |  |  |
|  | **3 months** | 9.3 | 18.0 | 8.1 | 4.301 | 11.851 | **<0.001** | **0.006** |
|  | **6 months** | 14.9 | 19.5 | 2.5 | -1.502 | 6.454 | 0.222 | 41.292 |
|  | **9 months** | 16.1 | 19.4 | 1.3 | -2.765 | 5.446 | 0.521 | 96.906 |
|  | **12 months** | 15.3 | 20.8 | 2.1 | -2.490 | 6.637 | 0.372 | 69.192 |
|  | **18 months** | 13.5 | 17.5 | 4.0 | -0.490 | 8.399 | 0.081 | 15.066 |
|  | **24 months** | 12.0 | 15.1 | 5.4 | 0.290 | 10.541 | **0.039** | 7.254 |
| **Anxiety** | **Baseline** | 48.9 | 25.3 |  |  |  |  |  |
|  | **3 months** | 41.3 | 27.2 | 7.6 | 2.868 | 12.353 | **0.002** | 0.372 |
|  | **6 months** | 32.7 | 25.2 | 16.1 | 11.467 | 20.830 | **<0.001** | **<0.001** |
|  | **9 months** | 29.0 | 26.8 | 19.9 | 14.869 | 24.853 | **<0.001** | **<0.001** |
|  | **12 months** | 30.8 | 26.6 | 18.1 | 12.933 | 23.273 | **<0.001** | **<0.001** |
|  | **18 months** | 27.2 | 27.3 | 21.6 | 15.755 | 27.519 | **<0.001** | **<0.001** |
|  | **24 months** | 22.0 | 21.4 | 26.9 | 19.375 | 34.387 | **<0.001** | **<0.001** |
| **Eating with others** | **Baseline** | 14.0 | 26.6 |  |  |  |  |  |
|  | **3 months** | 10.8 | 23.0 | 3.3 | -1.254 | 7.755 | 0.157 | 29.202 |
|  | **6 months** | 16.6 | 25.3 | -2.5 | -7.406 | 2.317 | 0.304 | 56.544 |
|  | **9 months** | 12.4 | 24.1 | 1.6 | -3.350 | 6.568 | 0.524 | 97.464 |
|  | **12 months** | 10.0 | 21.1 | 4.0 | -0.626 | 8.697 | 0.090 | 16.740 |
|  | **18 months** | 9.5 | 19.5 | 4.5 | -0.418 | 9.441 | 0.073 | 13.578 |
|  | **24 months** | 9.3 | 19.1 | 4.7 | -1.567 | 10.971 | 0.140 | 26.040 |
| **Dry mouth** | **Baseline** | 13.8 | 21.5 |  |  |  |  |  |
|  | **3 months** | 21.1 | 28.4 | -7.2 | -11.889 | -2.604 | **0.002** | 0.372 |
|  | **6 months** | 25.7 | 29.7 | -11.9 | -16.858 | -6.998 | **<0.001** | **<0.001** |
|  | **9 months** | 21.8 | 27.0 | -8.0 | -12.778 | -3.139 | **0.001** | 0.186 |
|  | **12 months** | 20.0 | 25.9 | -6.2 | -11.096 | -1.292 | **0.013** | 2.418 |
|  | **18 months** | 20.6 | 26.3 | -6.8 | -12.523 | -1.135 | **0.019** | 3.534 |
|  | **24 months** | 16.7 | 20.5 | -2.9 | -9.329 | 3.608 | 0.385 | 71.610 |
| **Trouble with taste** | **Baseline** | 12.4 | 24.2 |  |  |  |  |  |
|  | **3 months** | 22.3 | 29.2 | -10.0 | -14.921 | -5.020 | **<0.001** | 0.070 |
|  | **6 months** | 25.7 | 31.4 | -13.3 | -18.665 | -8.005 | **<0.001** | **<0.001** |
|  | **9 months** | 18.3 | 24.4 | -6.0 | -10.673 | -1.294 | **0.013** | 2.418 |
|  | **12 months** | 14.0 | 22.9 | -1.6 | -6.406 | 3.125 | 0.499 | 92.814 |
|  | **18 months** | 14.3 | 26.1 | -1.9 | -7.533 | 3.680 | 0.500 | 93.000 |
|  | **24 months** | 14.7 | 23.5 | -2.3 | -9.613 | 4.999 | 0.535 | 99.51 |
| **Trouble swallowing saliva** | **Baseline** | 9.5 | 19.9 |  |  |  |  |  |
|  | **3 months** | 14.7 | 24.0 | -5.2 | -9.225 | -1.145 | **0.012** | 2.232 |
|  | **6 months** | 13.9 | 23.4 | -4.4 | -8.507 | -0.351 | **0.033** | 6.138 |
|  | **9 months** | 12.5 | 22.0 | -3.1 | -7.156 | 1.035 | 0.142 | 26.412 |
|  | **12 months** | 14.2 | 26.0 | -4.7 | -9.553 | 0.084 | 0.054 | 10.044 |
|  | **18 months** | 13.0 | 24.7 | -3.5 | -8.855 | 1.799 | 0.193 | 35.898 |
|  | **24 months** | 10.0 | 18.1 | -0.5 | -6.453 | 5.429 | 0.866 | 161.076 |
| **Choked when swallowing** | **Baseline** | 7.7 | 18.0 |  |  |  |  |  |
|  | **3 months** | 8.0 | 18.2 | -0.3 | -3.541 | 3.015 | 0.875 | 162.750 |
|  | **6 months** | 14.2 | 23.0 | -6.5 | -10.440 | -2.610 | **0.001** | 0.186 |
|  | **9 months** | 12.9 | 22.1 | -5.2 | -9.205 | -1.254 | **0.010** | 1.860 |
|  | **12 months** | 10.3 | 18.5 | -2.6 | -6.268 | 1.109 | 0.170 | 31.620 |
|  | **18 months** | 12.8 | 20.4 | -5.1 | -9.616 | -0.602 | **0.027** | 5.022 |
|  | **24 months** | 12.2 | 20.1 | -4.5 | -10.667 | 1.600 | 0.145 | 26.970 |
| **Trouble with coughing** | **Baseline** | 21.8 | 23.0 |  |  |  |  |  |
|  | **3 months** | 27.7 | 29.9 | -5.9 | -10.807 | -0.946 | **0.020** | 3.720 |
|  | **6 months** | 43.5 | 30.1 | -21.7 | -26.772 | -16.551 | **<0.001** | **<0.001** |
|  | **9 months** | 35.3 | 26.5 | -13.5 | -18.189 | -8.750 | **<0.001** | **<0.001** |
|  | **12 months** | 26.2 | 23.4 | -4.4 | -9.010 | 0.261 | 0.064 | 11.904 |
|  | **18 months** | 28.9 | 23.6 | -7.0 | -12.285 | -1.798 | **0.009** | 1.674 |
|  | **24 months** | 26.5 | 21.5 | -4.7 | -11.639 | 2.274 | 0.186 | 34.596 |
| **Trouble talking** | **Baseline** | 4.8 | 14.3 |  |  |  |  |  |
|  | **3 months** | 9.7 | 23.0 | -4.9 | -8.468 | -1.325 | **0.007** | 1.302 |
|  | **6 months** | 15.5 | 27.3 | -10.6 | -14.856 | -6.382 | **<0.001** | **<0.001** |
|  | **9 months** | 11.0 | 23.8 | -6.2 | -10.203 | -2.222 | **0.002** | 0.372 |
|  | **12 months** | 8.7 | 19.9 | -3.8 | -7.460 | -0.208 | **0.038** | 7.068 |
|  | **18 months** | 8.6 | 20.7 | -3.7 | -8.085 | 0.608 | 0.091 | 16.926 |
|  | **24 months** | 5.3 | 12.3 | -0.5 | -4.748 | 3.747 | 0.817 | 151.962 |
| **Weight loss** | **Baseline** | 17.5 | 25.1 |  |  |  |  |  |
|  | **3 months** | 17.3 | 24.9 | 0.2 | -4.311 | 4.770 | 0.921 | 171.306 |
|  | **6 months** | 22.6 | 26.0 | -5.0 | -9.759 | -0.305 | **0.037** | 6.882 |
|  | **9 months** | 23.6 | 27.9 | -6.1 | -11.265 | -0.882 | **0.022** | 4.092 |
|  | **12 months** | 20.0 | 26.8 | -2.5 | -7.609 | 2.684 | 0.347 | 64.542 |
|  | **18 months** | 14.3 | 25.3 | 3.3 | -2.433 | 8.936 | 0.261 | 48.546 |
|  | **24 months** | 15.0 | 26.4 | 2.6 | -5.151 | 10.294 | 0.513 | 95.418 |
| **Problems with hair loss** | **Baseline** | 86.6 | 62.3 |  |  |  |  |  |
|  | **3 months** | 47.3 | 54.6 | 39.3 | 24.272 | 54.251 | **<0.001** | **<0.001** |
|  | **6 months** | 60.4 | 59.8 | 26.2 | 9.733 | 42.721 | **0.002** | 0.372 |
|  | **9 months** | 63.2 | 60.9 | 23.4 | 5.234 | 41.479 | **0.012** | 2.232 |
|  | **12 months** | 66.2 | 62.6 | 20.4 | 1.491 | 39.337 | **0.035** | 6.510 |
|  | **18 months** | 62.7 | 64.1 | 23.9 | 2.670 | 45.205 | **0.028** | 5.208 |
|  | **24 months** | 81.9 | 62.9 | 4.7 | -23.213 | 32.533 | 0.741 | 137.826 |

_B = regression coefficient. 95% CI = confidence interval. Bold p values represent significance (p<0.05). Corrected p value = corrected according to the Bonferroni correction for multiple testing._

### Supplementary table 5: Linear mixed models analysis of health-related quality of life scores at baseline and at 3, 6, 9, 12, 18 and 24 months follow-up including uncorrected p values in patients with grade 1 or no anastomotic leakage (-) and patients with grade 2 or 3 anastomotic leakage (+) following esophagectomy.

|  |  | **Mean HR-QoL score** | | | | | | |  |  |
| --- | --- | --- | --- | --- | --- | --- | --- | --- | --- | --- |
|  |  | **Baseline** | **3 months** | **6 months** | **9 months** | **12 months** | **18 months** | **24 months** | **p value** | **Corrected p value** |
|  | **N(-)** | **432** | **358** | **342** | **302** | **271** | **189** | **107** |  |  |
|  | **N(+)** | **54** | **40** | **36** | **29** | **25** | **15** | **6** |  |  |
| **EORTC QLQ-C30** | |  |  |  |  |  |  |  |  |  |
| **Global Health** | **-** | 74.3 | 71.0 | 69.2 | 73.2 | 73.6 | 73.7 | 75.7 | 0.097 | 3.007 |
|  | **+** | 74.9 | 68.6 | 65.2 | 69.6 | 69.8 | 69.5 | 67.3 |  |  |
| **Functioning scores** | |  |  |  |  |  |  |  |  |  |
| **Physical functioning** | **-** | 88.4 | 79.5 | 77.2 | 81.1 | 81.2 | 82.2 | 82.7 | **<0.001** | 0.009 |
|  | **+** | 88.4 | 67.5 | 65.1 | 72.4 | 75.3 | 75.0 | 68.4 |  |  |
| **Role functioning** | **-** | 81.1 | 67.6 | 65.2 | 72.6 | 75.5 | 76.5 | 75.6 | 0.005 | 0.155 |
|  | **+** | 81.2 | 63.2 | 43.9 | 62.2 | 69.1 | 75.6 | 52.4 |  |  |
| **Emotional functioning** | **-** | 78.9 | 81.9 | 84.4 | 84.2 | 84.7 | 83.4 | 85.0 | 0.102 | 3.162 |
|  | **+** | 74.8 | 80.6 | 78.8 | 78.2 | 78.4 | 81.2 | 81.7 |  |  |
| **Cognitive functioning** | **-** | 89.0 | 86.5 | 85.8 | 85.1 | 85.8 | 85.1 | 84.6 | 0.380 | 11.780 |
|  | **+** | 89.8 | 80.8 | 81.3 | 81.9 | 84.0 | 86.6 | 82.0 |  |  |
| **Social functioning** | **-** | 84.9 | 76.9 | 75.1 | 79.8 | 84.3 | 82.7 | 85.2 | 0.026 | 0.806 |
|  | **+** | 84.6 | 72.8 | 65.0 | 67.6 | 79.0 | 74.4 | 80.7 |  |  |
| **Symptom scores** | |  |  |  |  |  |  |  |  |  |
| **Fatigue** | **-** | 25.5 | 34.2 | 36.6 | 32.7 | 30.8 | 30.3 | 29.4 | 0.008 | 0.248 |
|  | **+** | 29.2 | 45.8 | 49.4 | 40.5 | 33.7 | 36.4 | 38.6 |  |  |
| **Nausea and vomiting** | **-** | 11.2 | 11.3 | 17.2 | 14.2 | 10.7 | 9.8 | 9.7 | 0.520 | 16.120 |
|  | **+** | 10.5 | 14.5 | 19.1 | 18.1 | 10.5 | 9.9 | 10.8 |  |  |
| **Pain** | **-** | 15.7 | 20.0 | 18.1 | 16.4 | 16.2 | 14.0 | 14.9 | 0.039 | 1.209 |
|  | **+** | 15.1 | 24.7 | 23.7 | 21.3 | 16.9 | 23.3 | 29.4 |  |  |
| **Dyspnea** | **-** | 11.6 | 20.0 | 26.2 | 20.0 | 18.9 | 19.0 | 16.6 | **<0.001** | 0.008 |
|  | **+** | 13.6 | 31.8 | 40.6 | 32.3 | 28.5 | 30.3 | 38.2 |  |  |
| **Insomnia** | **-** | 23.4 | 26.7 | 22.8 | 19.7 | 22.0 | 21.6 | 20.7 | 0.468 | 14.508 |
|  | **+** | 24.1 | 32.6 | 28.5 | 18.8 | 21.8 | 24.5 | 24.3 |  |  |
| **Appetite loss** | **-** | 19.0 | 24.8 | 35.0 | 24.0 | 20.2 | 18.7 | 16.1 | 0.312 | 9.672 |
|  | **+** | 13.0 | 25.5 | 37.8 | 21.4 | 14.7 | 11.3 | 10.9 |  |  |
| **Constipation** | **-** | 14.1 | 13.5 | 11.0 | 11.1 | 9.7 | 8.7 | 11.3 | 0.775 | 24.025 |
|  | **+** | 11.1 | 18.1 | 15.1 | 8.0 | 8.7 | 12.0 | 11.2 |  |  |
| **Diarrhea** | **-** | 6.2 | 11.0 | 21.0 | 16.7 | 15.3 | 14.2 | 14.1 | **0.001** | 0.031 |
|  | **+** | 11.1 | 19.2 | 29.4 | 25.7 | 20.3 | 19.0 | 39.2 |  |  |
| **Financial difficulties** | **-** | 5.6 | 7.5 | 6.7 | 7.2 | 7.3 | 6.6 | 5.1 | 0.110 | 3.410 |
|  | **+** | 9.3 | 10.8 | 11.4 | 12.2 | 9.4 | 12.6 | 6.3 |  |  |
| **EORTC QLQ-OG25** | |  |  |  |  |  |  |  |  |  |
| **Functioning** | |  |  |  |  |  |  |  |  |  |
| **Body image** | **-** | 90.9 | 86.1 | 84.2 | 87.5 | 86.5 | 86.3 | 89.6 | 0.159 | 4.929 |
|  | **+** | 90.1 | 85.6 | 86.6 | 83.7 | 77.4 | 80.8 | 79.3 |  |  |
| **Symptom scores** | |  |  |  |  |  |  |  |  |  |
| **Dysphagia** | **-** | 22.1 | 18.1 | 19.8 | 14.7 | 13.7 | 11.5 | 9.4 | 0.085 | 2.635 |
|  | **+** | 17.0 | 23.0 | 33.8 | 24.6 | 18.3 | 13.8 | 5.6 |  |  |
| **Eating** | **-** | 32.2 | 29.0 | 38.8 | 31.1 | 28.9 | 26.1 | 24.6 | 0.128 | 3.968 |
|  | **+** | 25.1 | 36.6 | 48.0 | 40.5 | 33.5 | 32.9 | 27.0 |  |  |
| **Reflux** | **-** | 6.9 | 7.4 | 13.7 | 15.1 | 16.0 | 17.8 | 14.7 | 0.652 | 20.212 |
|  | **+** | 5.2 | 7.5 | 15.6 | 16.9 | 10.7 | 13.3 | 14.3 |  |  |
| **Odynophagia** | **-** | 24.5 | 15.4 | 15.0 | 12.1 | 10.8 | 9.5 | 7.5 | 0.217 | 6.727 |
|  | **+** | 21.6 | 15.8 | 22.2 | 17.1 | 12.2 | 14.5 | 8.5 |  |  |
| **Pain and discomfort** | **-** | 16.2 | 11.6 | 15.1 | 15.8 | 15.8 | 14.5 | 12.8 | 0.698 | 21.638 |
|  | **+** | 22.5 | 12.3 | 15.1 | 19.2 | 11.9 | 6.2 | 8.0 |  |  |
| **Anxiety** | **-** | 50.1 | 40.7 | 32.1 | 29.6 | 28.8 | 27.3 | 26.4 | 0.868 | 26.908 |
|  | **+** | 47.2 | 41.4 | 32.4 | 30.2 | 30.5 | 30.8 | 26.4 |  |  |
| **Eating with others** | **-** | 14.5 | 10.5 | 15.1 | 12.5 | 11.2 | 10.2 | 7.2 | 0.090 | 2.790 |
|  | **+** | 14.2 | 13.8 | 16.1 | 13.8 | 11.3 | 13.8 | 30.0 |  |  |
| **Dry mouth** | **-** | 13.1 | 19.0 | 25.6 | 20.9 | 20.1 | 20.3 | 21.8 | 0.808 | 25.048 |
|  | **+** | 14.2 | 27.0 | 25.4 | 19.2 | 16.2 | 11.7 | 21.2 |  |  |
| **Trouble with taste** | **-** | 11.7 | 20.0 | 24.2 | 17.1 | 16.5 | 14.1 | 14.7 | 0.817 | 25.327 |
|  | **+** | 6.2 | 31.8 | 24.6 | 18.1 | 12.0 | 9.4 | 21.2 |  |  |
| **Trouble swallowing saliva** | **-** | 9.1 | 13.9 | 15.8 | 12.5 | 13.5 | 13.7 | 10.4 | 0.159 | 4.929 |
|  | **+** | 9.9 | 14.4 | 13.4 | 16.3 | 22.6 | 19.2 | 20.7 |  |  |
| **Choked when swallowing** | **-** | 7.0 | 7.8 | 11.4 | 8.7 | 8.2 | 10.2 | 9.6 | **<0.001** | **<0.001** |
|  | **+** | 9.9 | 12.6 | 26.5 | 28.5 | 20.7 | 14.3 | 21.6 |  |  |
| **Trouble with coughing** | **-** | 20.1 | 27.6 | 41.2 | 32.6 | 26.6 | 27.4 | 23.8 | 0.003 | 0.093 |
|  | **+** | 25.3 | 36.3 | 57.2 | 44.8 | 35.1 | 36.7 | 29.3 |  |  |
| **Trouble talking** | **-** | 4.6 | 9.9 | 13.6 | 8.1 | 7.6 | 6.6 | 4.0 | 0.199 | 6.169 |
|  | **+** | 4.3 | 11.0 | 18.9 | 13.0 | 12.8 | 16.7 | -4.8 |  |  |
| **Weight loss** | **-** | 17.9 | 18.2 | 23.5 | 22.0 | 19.9 | 16.3 | 16.9 | 0.253 | 7.843 |
|  | **+** | 19.5 | 22.0 | 22.0 | 29.7 | 24.1 | 16.8 | 27.0 |  |  |
| **Problems with hair loss** | **-** | 76.5 | 48.7 | 57.5 | 53.3 | 62.7 | 60.6 | 63.1 | 0.758 | 23.498 |
|  | **+** | 91.5 | 29.6 | 42.5 | 73.6 | 28.3 | 40.3 | 93.6 |  |  |

_N (+) = number of patients with grade 2-3 anastomotic leakage, N (-) = number of patients with grade 1 or no anastomotic leakage. Values are represented as mean HR-QoL scores. P value = p value over time. Corrected p value = p value over time that is corrected for multiple testing according to Bonferroni method. Bold p values represent significance (p<0.001)._
